# Portuguese-Brazilian evidence-based guideline on the management of hyperglycemia in type 2 diabetes mellitus

**DOI:** 10.1186/s13098-020-00551-1

**Published:** 2020-05-24

**Authors:** Marcello Casaccia Bertoluci, João Eduardo Nunes Salles, José Silva-Nunes, Hermelinda Cordeiro Pedrosa, Rodrigo Oliveira Moreira, Rui Manuel Calado da Silva Duarte, Davide Mauricio da Costa Carvalho, Fábio Rogério Trujilho, João Filipe Cancela dos Santos Raposo, Erika Bezerra Parente, Fernando Valente, Fábio Ferreira de Moura, Alexandre Hohl, Miguel Melo, Francisco Garcia Pestana Araujo, Rosa Maria Monteiro Castro de Araújo Principe, Rosane Kupfer, Adriana Costa e Forti, Cynthia Melissa Valerio, Hélder José Ferreira, João Manuel Sequeira Duarte, José Francisco Kerr Saraiva, Melanie Rodacki, Maria Helane Costa Gurgel Castelo, Mariana Pereira Monteiro, Patrícia Quadros Branco, Pedro Manuel Patricio de Matos, Pedro Carneiro de Melo Pereira de Magalhães, Roberto Tadeu Barcellos Betti, Rosângela Roginski Réa, Thaisa Dourado Guedes Trujilho, Lana Catani Ferreira Pinto, Cristiane Bauermann Leitão

**Affiliations:** 1 Internal Medicine Department, School of Medicine, Universidade Federal do Rio Grande do Sul (UFRGS), Rua Ramiro Barcelos, 2350, 4º Andar, Porto Alegre, RS 90035-007 Brazil; 2 Endocrinology Unit, Hospital de Clínicas de Porto Alegre (HCPA-UFRGS), Rua Ramiro Barcelos, 2350, 4º Andar, Porto Alegre, RS 90035-007 Brazil; 3 Department of Internal Medicine, Discipline of Endocrinology, Faculdade de Ciências Médicas da Santa Casa de São Paulo (FCMSCSP), Rua Dr. Cesário Mota Junior, 61, São Paulo, SP 01221-020 Brazil; 4 Department of Endocrinology, Diabetes and Metabolism/Centro Hospitalar, Universitário de Lisboa Central (CHULC), Rua da Beneficência, 8, 1069-166 Lisbon, Portugal; 5 NOVA Medical School (NMS)/Faculdade de Ciências Médicas (FCM) da Universidade Nova de Lisboa, Rua da Beneficência, 8, 1069-166 Lisbon, Portugal; 6 Health and Technology Research Center/Escola Superior de Tecnologia da Saúde de Lisboa, Rua da Beneficência, 8, 1069-166 Lisbon, Portugal; 7 Hospital Curry Cabral, Rua da Beneficência, 8, 1069-166 Lisbon, Portugal; 8 Endocrinology Unit and Research Centre, Hospital Regional de Taguatinga, Área Especial Nº 24, Setor C Norte, Taguatinga Norte, Brasília, DF 72115-920 Brazil; 9 Instituto Estadual de Diabetes e Endocrinologia Luiz Capriglione (IEDE), Rua Moncorvo Filho, 90, Rio de Janeiro, RJ 20211-340 Brazil; 10 Faculdade de Medicina, Universidade Presidente Antônio Carlos (UNIPAC), Juiz de Fora, MG Brazil; 11 Centro Universitário de Valença (UNIFAA), Rua Moncorvo Filho, 90, Rio de Janeiro, RJ 20211-340 Brazil; 12 Associação Protetora dos Diabéticos de Portugal (APDP), Rua Rodrigo da Fonseca 1, 1250-189 Lisbon, Portugal; 13 Department of Endorinology, Diabetes and Metabolism, Centro Hospitalar S. João, Porto, Portugal; 14 Faculty of Medicine, i3S, Universidade do Porto, Porto, Portugal; 15 Department of Obesity, Sociedade Brasileira de Endocrinologia e Metabologia, Av. Antonio Carlos Magalhães, s/n, Parque Bela Vista, Salvador, BA 40275-350 Brazil; 16 NOVA Medical School (NMS), Faculdade de Ciências Médicas (FCM), Universidade Nova de Lisboa, Rua Salitre, 118, 1250-203 Lisbon, Portugal; 17 Associação Protetora dos Diabéticos de Portugal (APDP), Rua Salitre, 118, 1250-203 Lisbon, Portugal; 18 Sociedade Portuguesa de Diabetologia (SPD), Rua Salitre, 118, 1250-203 Lisbon, Portugal; 19 Department of Endocrinology, Faculdade de Ciências Médicas da Santa Casa de São Paulo (FCMSCSP), Rua Dr. Cesario Mota Jr., 112, São Paulo, SP 01221-010 Brazil; 20 Endocrinology Division, Department of Internal Medicine, Faculdade de Medicina do ABC, Av. Lauro Gomes, 2000, Santo André, SP Brazil; 21 Department of Endocrinology, Universidade de Pernambuco (UPE), Rua Arnobio Marques, 310, Recife, PE 50100-130 Brazil; 22 Endocrinology Service, Instituto de Medicina de Pernambuco (IMIP), Rua Arnobio Marques, 310, Recife, PE 50100-130 Brazil; 23 Department of Endocrinology and Metabolism/Department of Internal Medicine, Universidade Federal de Santa Catarina (UFSC), Rua Professora Maria Flora Pausewang, s/n, Florianópolis, SC 88036-800 Brazil; 24 Hospital Universitário Polydoro Ernani de São Thiago, Campus Universitário, Rua Professora Maria Flora Pausewang, s/n, Florianópolis, SC 88036-800 Brazil; 25 Department of Endocrinology, Diabetes and Metabolism, Centro Hospitalar e Universitário de Coimbra, Coimbra, Portugal; 26 Medical Faculty, University of Coimbra, Praceta Mota Pinto, 3000-075 Coimbra, Portugal; 27 Serviço de Medicina, Hospital Beatriz Angelo, Loures, Portugal; 28 Endocrinology Service, Hospital Pedro Hispano, Unidade Local de Saúde de Matosinhos, Rua Dr. Eduardo Torres, 4464/513 Senhora da Hora, Portugal; 29 Department of Diabetes, Instituto Estadual de Diabetes e Endocrinologia Luiz Capriglione (IEDE), Rua Moncorvo Filho, 90, Rio de Janeiro, RJ 20211-340 Brazil; 30 Department of Internal Medicine, School of Medicine, Universidade Federal do Ceará (UFC), Rua Capitão Francisco Pedro, 1290, Fortaleza, CE 60430-375 Brazil; 31 Unidade de Saúde Familiar Coimbra Celas, Administração Regional de Saúde do Centro, Av. D. Afonso Henriques, 141, 3000-011 Coimbra, Portugal; 32 Endocrinology Service, Hospital Egas Moniz, Rua Junqueira, 126, 1349-019 Lisbon, Portugal; 33 Cardiology Division, Faculdade de Medicina, Pontifícia Universidade Católica de Campinas (PUC-Campinas), Rua Engenheiro Carlos Stevenson, 560, Campinas, SP 13092-132 Brazil; 34 Instituto de Pesquisa Clínica de Campinas (IPECC), Rua Engenheiro Carlos Stevenson, 560, Campinas, SP 13092-132 Brazil; 35 Department of Internal Medicine, Diabetes and Nutrology Section, Universidade Federal do Rio de Janeiro (UFRJ), Rua Rodolpho Paulo Rocco. 255, Sala 9E14, Rio de Janeiro, RJ Brazil; 36 Universidade Federal do Ceará (UFC), Rua Capitão Francisco Pedro, 1290, Fortaleza, CE 60430-375 Brazil; 37 Unidade de Investigação Multidisciplicar Biomédica, Instituto de Ciências Biomédicas de Abel Salazar, Universidade do Porto, Porto, Portugal; 38 Nephrology Service, Centro Hospitalar Lisboa Ocidental, Rua Rodrigo da Fonseca, 1, 1250-189 Lisbon, Portugal; 39 Diretoria Clínica, Nephrocare, Rua Rodrigo da Fonseca, 1, 1250-189 Lisbon, Portugal; 40 Department of Cardiology, Associação Protetora dos Diabéticos de Portugal (APDP), Rua Rodrigo da Fonseca, 1250, 189, Lisbon, Portugal; 41 Endocrinology Service, Hospital Pedro Hispano, Rua Dr. Eduardo Torres, 4464/513 Senhora da Hora, Portugal; 42 Hospital Alemão Oswaldo Cruz, Centro de Obesidade e Diabetes, Rua 13 de Maio, 1815, São Paulo, SP 01327-001 Brazil; 43 Department of Internal Medicine, Serviço de Endocrinologia e Metabologia, Hospital de Clínicas, Universidade Federal do Paraná (UFPR), Av. Agostinho Leão Junior, 285, Curitiba, PR 80030-110 Brazil; 44 Department of Diabetes Mellitus, Sociedade Brasileira de Endocrinologia e Metabologia, Av. Antonio Carlos Magalhães, s/n, Salvador, BA 40275-350 Brazil; 45 Sociedade Brasileira de Diabetes, Regional Bahia, Av. Antonio Carlos Magalhães, s/n, Salvador, BA 40275-350 Brazil

**Keywords:** Diabetes treatment, Type 2 diabetes, Cardiovascular risk, Guidelines, Heart failure, Chronic kidney disease, Ischemic heart disease, ASCVD, Atherosclerotic disease

## Abstract

**Background:**

In current management of type 2 diabetes (T2DM), cardiovascular and renal prevention have become important targets to be achieved. In this context, a joint panel of four endocrinology societies from Brazil and Portugal was established to develop an evidence-based guideline for treatment of hyperglycemia in T2DM.

**Methods:**

MEDLINE (via PubMed) was searched for randomized clinical trials, meta-analyses, and observational studies related to diabetes treatment. When there was insufficient high-quality evidence, expert opinion was sought. Updated positions on treatment of T2DM patients with heart failure (HF), atherosclerotic CV disease (ASCVD), chronic kidney disease (CKD), and patients with no vascular complications were developed. The degree of recommendation and the level of evidence were determined using predefined criteria.

**Results and conclusions:**

In non-pregnant adults, the recommended HbA_1c_ target is below 7%. Higher levels are recommended in frail older adults and patients at higher risk of hypoglycemia. Lifestyle modification is recommended at all phases of treatment. Metformin is the first choice when HbA_1c_ is 6.5–7.5%. When HbA_1c_ is 7.5–9.0%, dual therapy with metformin plus an SGLT2i and/or GLP-_1_RA (first-line antidiabetic agents, AD1) is recommended due to cardiovascular and renal benefits. If an AD1 is unaffordable, other antidiabetic drugs (AD) may be used. Triple or quadruple therapy should be considered when HbA_1c_ remains above target. In patients with clinical or subclinical atherosclerosis, the combination of one AD1 plus metformin is the recommended first-line therapy to reduce cardiovascular events and improve blood glucose control. In stable heart failure with low ejection fraction (< 40%) and glomerular filtration rate (eGFR) > 30 mL/min/1.73 m^2^, metformin plus an SGLT-2i is recommended to reduce cardiovascular mortality and heart failure hospitalizations and improve blood glucose control. In patients with diabetes-associated chronic kidney disease (CKD) (eGFR 30–60 mL/min/1.73 m^2^ or eGFR 30–90 mL/min/1.73 m^2^ with albuminuria > 30 mg/g), the combination of metformin and an SGLT2i is recommended to attenuate loss of renal function, reduce albuminuria and improve blood glucose control. In patients with severe renal failure, insulin-based therapy is recommended to improve blood glucose control. Alternatively, GLP-_1_RA, DPP4i, gliclazide MR and pioglitazone may be considered to reduce albuminuria. In conclusion, the current evidence supports individualizing anti-hyperglycemic treatment for T2DM.

## Background

As new medications have become available in recent years, perspectives on the management of people with type 2 diabetes (T2DM) have evolved into a broader approach in which primary and secondary cardiovascular and renal prevention have become important targets. The unique characteristics of these new antidiabetic agents, with proven cardiovascular (CV) and renal benefits, have compelled scientific societies to update their guidelines. In this line, the present guideline is a joint initiative of four societies from Portugal and Brazil: *Sociedade Brasileira de Diabetes* (SBD), *Sociedade Brasileira de Endocrinologia e Metabologia* (SBEM), *Sociedade Portuguesa de Diabetologia* (SPD) and *Sociedade Portuguesa de Endocrinologia, Diabetes, e Metabolismo* (SPEDM).

To develop this guideline, the best evidence available was reviewed and the expert opinions of a Portuguese-Brazilian panel of diabetes specialists were obtained. A list of statements was carefully created and scored. When high-quality evidence was not available from the literature, the panel gave opinions on a variety of clinical scenarios. These opinions were captured and analyzed by an international voting system, which allowed consensus to be reached after multiple rounds of discussion. The main objective of this guideline is to support the decision-making process in clinical practice, taking into account patients’ best interests and clinicians’ personal preferences.

## Methods

The scientific societies appointed 33 specialists with extensive expertise in diabetes to compose the panel. The main clinical topics requiring updated positions in patients with T2DM were heart failure (HF), atherosclerotic CV disease (ASCVD), chronic kidney disease (CKD), and management of T2DM in patients without vascular complications. The panel compiled a narrative review by searching MEDLINE (via PubMed) for randomized clinical trials, meta-analyses, and high-quality observational studies related to type 2 diabetes treatment, using the MeSH terms [diabetes], [type 2 diabetes], [cardiovascular disease], [coronary artery disease], [heart failure], and [chronic kidney disease]. When the results of the search did not yield enough high-quality evidence to answer a specific question or scenario, an expert opinion was sought: a query was sent to all panelists, and responses were recorded. The frequency of responses was analyzed and a consensus opinion was drawn up.

The degree of recommendation depended on the query, following the criteria that are shown in Table 1A. The level of evidence was determined using the same criteria in Table 1B. Specific criteria for atherosclerotic cardiovascular disease (ASCVD) are shown in Table 2 [1]. The panel chose to classify therapeutic options into two groups of glucose-lowering agents: antidiabetics with proven CV or renal benefit (AD1) and general glucose-lowering agents (AD). These are specified in Table 3.

**Table 1:**
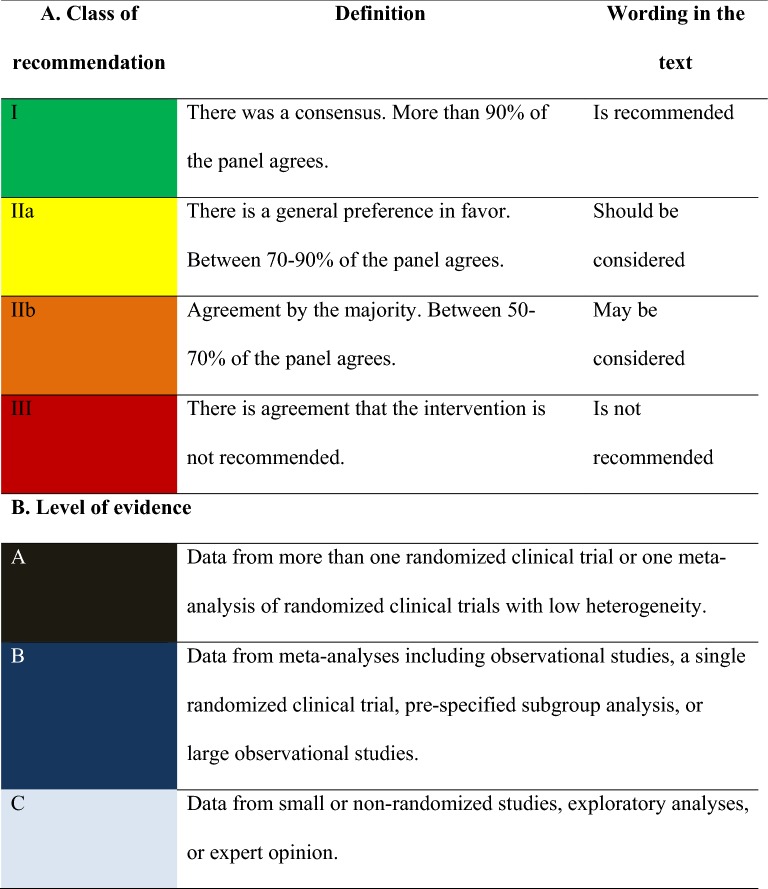
Class of recommendation and level of evidence

**Table 2:** Definitions of atherosclerotic cardiovascular disease (ASCVD) [1]

Clinical atherosclerosis	
Acute coronary syndrome: acute myocardial infarction and/or unstable angina Stable angina or previous acute myocardial infarction Atherothrombotic stroke or transient ischemic attack Coronary, carotid, renal-artery, or peripheral revascularization Peripheral vascular insufficiency or limb amputation Severe atherosclerotic disease (stenosis > 50%) in any vascular territory

**Table 3:**
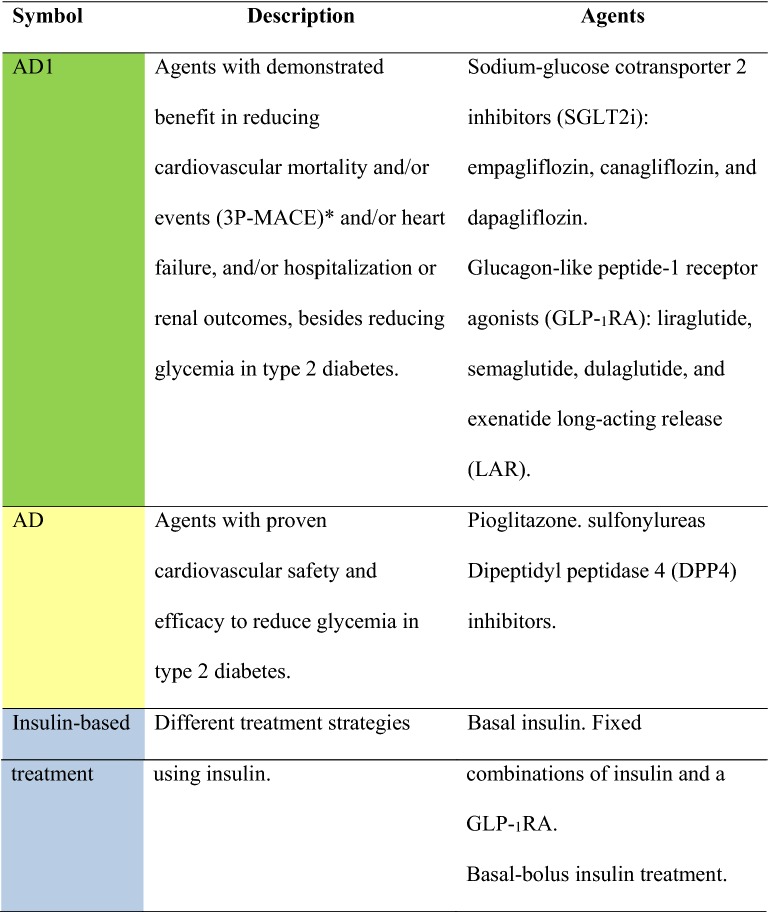
Types of antidiabetic agents

^a^3P-MACE: composite of three major adverse cardiovascular events (nonfatal myocardial infarction, nonfatal stroke, and cardiovascular death)

The guideline-building process was conducted as described elsewhere [1]. In brief, a preliminary manuscript outlining grades of recommendation and levels of evidence was drafted. Several rounds of discussion were held among the panel members, who reviewed the findings and made suggestions. The manuscript was then returned to the lead author for inclusion of changes. The same procedure was repeated for each of the four modules (ASCVD, HF, CKD, and patients without vascular complications). Subsequently, many other rounds of revision were done by request of subcommittee members. Then, the manuscript was presented for public consultation and discussed with the audience; minor adjustments were suggested. Finally, the consensus version of the document was submitted to the editorial board for final editing and proofreading.

## Recommendations and summary of evidence

### HbA_1C_ target

**1. In non-pregnant adult patients with T2DM, an HbA**_**1c**_**target of < 7% is recommended to reduce the incidence of microvascular complications.** 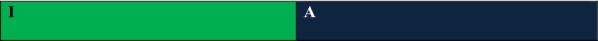


#### Summary of evidence

- The UKPDS 33 trial [2] showed that improving glucose control by reducing HbA_1c_ to a target of below 7% is clearly associated with reduced microvascular complications. A total of 3867 newly diagnosed patients with T2DM were randomly assigned to intensive treatment (sulfonylurea or insulin) or conventional treatment (diet alone). The aim in the intensive group was to attain a fasting plasma glucose (FPG) of less than 108 mg/dL, versus the best achievable FPG with diet alone in the conventional group. Three aggregate endpoints were considered: (1) any diabetes-related endpoint (sudden death, death from hyperglycemia or hypoglycemia, fatal or non-fatal myocardial infarction, angina, heart failure, stroke, renal failure, any amputation, vitreous hemorrhage, retinopathy requiring photocoagulation, blindness, or cataract extraction); (2) diabetes-related death (death from myocardial infarction, stroke, peripheral vascular disease, renal disease, hyperglycemia or hypoglycemia, and sudden death); and (3) all-cause mortality. After 10 years, the median HbA_1c_ was 7.0% (interquartile range [IQR], 6.2–8.2%) in the intensive group versus 7.9% (6.9–8.8%) in the conventional group. For any diabetes-related endpoint, risk was 12% lower in the intensive group (95% CI 1–21, *p* = 0.029) than in the conventional group. The risk reduction in the any diabetes-related composite endpoint was largely attributable to a 25% risk reduction (95% CI 7–40, *p* = 0.0099) in microvascular outcome events.
- Similar results were seen in the ADVANCE [3] study, which randomized 11,140 patients with T2DM to undergo either standard or intensive glucose control. After a median of 5 years of follow-up, the mean HbA_1c_ level in the intensive group was 6.5% compared to 7.3% in the standard-control group. Intensive blood glucose control also reduced the incidence of major microvascular events (new or worsening nephropathy or retinopathy) (HR, 0.86; 95% CI 0.77 to 0.97; *p* = 0.01).
- Although the landmark DCCT trial [4] was conducted in type 1 diabetes (T1DM), the panel considered that the effects of lowering blood glucose and HbA_1c_ down to 7% by an intensive glucose-lowering regimen with insulin reinforced the target level of < 7% in T2DM. In DCCT, 1441 patients with T1DM were randomized into intensive or conventional treatment with insulin-based therapy, targeting an HbA_1c_ of less than 6.05%. The HbA_1c_ attained in the intensive group was around 7%. In relation to conventional therapy, intensive therapy reduced retinopathy by 76% (primary prevention group) and 54% (secondary prevention group). Albuminuria was reduced by 39% (micro) and 54% (macro), whereas peripheral neuropathy was reduced by 60%.

**2. In non-pregnant adult patients with T2DM, an HbA**_**1c**_**target < 7% should be considered to reduce the long-term incidence of macrovascular complications.** 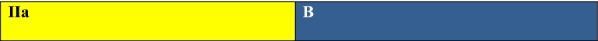


#### Summary of evidence

- Long-term post-trial observational follow-up studies [5] have shown that intensive blood glucose control can also decrease macrovascular complications. In the post-trial observational phase of UKPDS, 3277 patients with T2DM were followed for 5 years with no attempts to maintain previously assigned therapies. After the first year, no between-group differences in HbA_1c_ levels remained. In the sulfonylurea/insulin group, relative risk reductions persisted at 10 years for any diabetes-related endpoint (9%, *p* = 0.04) and for microvascular disease (24%, *p* = 0.001). However, there were also reductions in risk of myocardial infarction (15%, *p* = 0.01) and all-cause mortality (13%, *p* = 0.007). In the metformin subgroup, significant risk reductions persisted for any diabetes-related endpoint (21%, *p* = 0.01), myocardial infarction (33%, *p* = 0.005), and all-cause mortality (27%, *p* = 0.002).
- The Epidemiology of Diabetes Interventions and Complications (EDIC) study was a post-trial phase of the DCCT trial [6] in which 93% of DCCT survivors were followed. CV disease (defined as nonfatal myocardial infarction, stroke, death from CV disease, confirmed angina, or need for coronary-artery revascularization) was assessed with standardized measures. During a mean follow-up of 17 years, 46 CV disease events occurred in 31 patients who had received intensive treatment, versus 98 events in 52 patients who had received conventional treatment. Intensive treatment reduced the risk of any CV disease event by 42% (95% CI 9 to 63%; *p* = 0.02). The risk of nonfatal myocardial infarction, stroke, or CV death (3P-MACE) decreased by 57% (95% CI 12 to 79%; *p* = 0.02). The decrease in HbA_1c_ during the DCCT study was significantly associated with the positive effects of intensive treatment on the risk of CV disease. Intensive glucose control has long-term beneficial effects against CV risk in patients with T1DM as well.

**3. A higher individualized HbA**_**1c**_**target level is recommended in frail older adults, in the presence of comorbidities limiting life expectancy or when hypoglycemia is strongly to be avoided.** 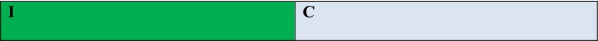


#### Summary of evidence

- This postulation was based on expert opinion. There was consensus among the panelists that HbA_1c_ targets should be higher in special clinical situations, to lower the risk of hypoglycemia.

**4. HbA**_**1c**_**measurements should be obtained at least once every 12** **weeks during treatment.**
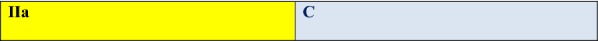


#### Summary of evidence

- This suggestion was based on the opinion and experience of the great majority of experts in the panel; no specific evidence was found. The objective is to monitor treatment effectiveness and improve adherence.

### Initial glucose lowering therapy in treatment-naïve patients with type 2 diabetes mellitus (T2DM) (Fig. 1)

**5. Lifestyle modification is recommended during all phases of treatment in T2DM to improve blood glucose control.** 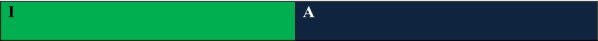

**Fig. 1:**
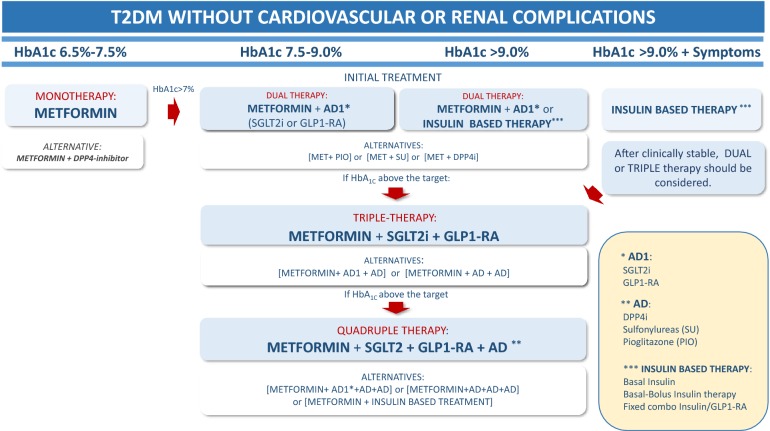
Decision support algorithm for treatment of hyperglycemia in the non-pregnant adult patient with type 2 diabetes mellitus

#### Summary of evidence

- Lifestyle measures should be recommended universally as the basis for diabetes treatment, as sustained remission of T2DM is related to the degree of weight loss. The DIRECT study [7] was an open-label, cluster-randomized, controlled trial conducted at primary health care units in the UK which randomized overweight/obese patients recently diagnosed with T2DM to an integrated structured weight management program (intervention) (*n* = 149) or the standard of care in accordance with UK guidelines (*n* = 149). The intervention included withdrawal of antidiabetic drugs, total diet replacement (825–853 kcal/day formula diet for 12–20 weeks) and stepped food reintroduction (2–8 weeks), followed by structured support for weight-loss maintenance. The main outcome was weight loss of at least 15 kg and remission of T2DM, defined as an HbA_1c_ < 6.5% after withdrawal of antidiabetic agents at 12 and 24 months. At 24 months, 11% of patients in the intervention group and 2% of controls had achieved weight loss of at least 15 kg (OR: 7.49; 95% CI 2.05 to 7.32; *p* = 0.0023), and remission of diabetes was seen in 36% in the intervention group and 3% in the control group (OR: 25.82; 95% CI 8.25 to 80.84; *p* < 0.0001). On post hoc analysis, among those patients who maintained at least 10 kg of weight loss (24% of those in the intervention group), 64% achieved remission.
- A meta-analysis [8] of randomized controlled trials (RCTs) assessed combinations of structured exercise regimens and physical activity advice with or without dietary co-intervention and their effects on change in HbA_1c_ in patients with T2DM. A total of 47 RCTs (duration ≥ 12 weeks) were included, for a total of 8538 patients. Structured exercise training was associated with a 0.67% reduction in HbA_1c_ (95% CI − 0.84 to − 0.49%) versus control. Structured exercise duration of > 150 min/week was associated with HbA_1c_ reductions of 0.89%. A combination of physical activity advice and dietary co-intervention was associated with a − 0.58% reduction in HbA_1c_ (95% CI − 0.74% to − 0.43%) compared with control.

**6. In treatment-naïve, non-pregnant adults recently diagnosed with T2DM, without cardiovascular or chronic renal disease, in whom HbA**_**1c**_**is 6.5–7.5%, METFORMIN monotherapy is recommended to improve blood glucose control and prevent diabetes-related outcomes.** 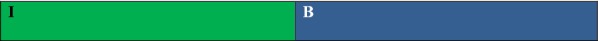


#### Summary of evidence

- Metformin is the first-line agent of choice for treatment of T2DM, given its established efficacy, safety profile, low incidence of hypoglycemia and low cost. The efficacy of metformin in reducing diabetes-related outcomes was demonstrated in overweight and obese patients in the UKDPS 34 trial [9]. The objective of UKPDS 34 was to investigate whether intensive blood-glucose control (treating to a fasting blood glucose of 108 mg/dL) with metformin, a sulfonylurea or insulin had any specific advantage or disadvantage. In a randomized controlled clinical trial, of 4075 patients recruited to UKPDS, 1704 overweight patients with newly diagnosed T2DM (with baseline fasting blood glucose 110–270 mg/dL) were assigned to either conventional treatment with diet alone (*n* = 411), intensive control with metformin (*n* = 342) or intensive control with a sulfonylurea or insulin (*n* = 951). The median duration was 10.7 years. The primary outcome measures were: any diabetes-related clinical endpoint, diabetes-related death, and all-cause mortality. Overall mean HbA_1c_ at baseline was 7.2 ± 1.5%. Compared with the conventional group, patients in the metformin group had risk reductions of 32% (95% CI 13–47, *p* = 0.002) for any diabetes-related endpoint, 42% for diabetes-related death (9–63, *p* = 0.017), and 36% for all-cause mortality (9–55, *p* = 0.011). Among patients allocated to intensive blood glucose control, metformin showed a greater effect than chlorpropamide, glibenclamide, or insulin for any diabetes-related endpoint (*p* = 0.0034), all-cause mortality (*p* = 0.021), and stroke (*p* = 0.032). Intensive glucose control with metformin decreased the risk of diabetes-related endpoints in overweight diabetic patients, and was associated with less weight gain and fewer hypoglycemic attacks than are insulin and sulfonylureas. It is thus the first-line pharmacological therapy of choice in these patients.
- Important note: This panel strongly recommends that, before initiating any treatment with antidiabetic agents, the eGFR should be estimated in every patient and the drug initiated only if in accordance with the product label. In the case of metformin, it should not be initiated when eGFR is below 45 mL/min/1.73 m^2^ and should be discontinued whenever eGFR is below 30 mL/min/1.73 m^2^, due to the risk of metabolic acidosis.

**7. In treatment-naïve, non-pregnant adults recently diagnosed with T2DM, without cardiovascular or chronic renal disease, in whom HbA**_**1c**_**is 6.5–7.5%, DUAL THERAPY, including metformin plus a DPP4i, may be considered to delay hyperglycemia treatment failure.** 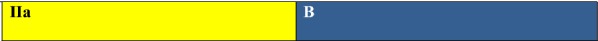


#### Summary of evidence

- The VERIFY study [10] was a randomized, multicenter, double-blind, parallel-group trial of newly diagnosed patients aged 18–70 years, diagnosed with T2DM within 2 years of enrollment, with HbA_1c_ levels between 6.5 and 7.5%. Patients were randomized to “early combination treatment” (metformin and vildagliptin) or “standard care” (metformin monotherapy plus placebo). If HbA_1c_ did not remain below 7.0% with initial treatment, patients in the metformin group were crossed over to vildagliptin instead of placebo, entering phase 2 of the study, during which all patients were given combination therapy. The primary efficacy endpoint was “time to initial treatment failure”, defined as HbA_1c_ ≥ 7.0% at two consecutive scheduled visits 13 weeks apart from randomization. A total of 2001 participants were randomized. The relative risk for time to failure was significantly lower in the early combination treatment group over the 5-year study period (HR 0.51; 95% CI 0.45 to 0.58; *p* < 0.0001). Both strategies were safe and well-tolerated. In patients with newly diagnosed T2DM, early intervention with combination therapy (metformin plus a DPP4i) appears to provides greater long-term glycemic control than metformin monotherapy. It is important to highlight that this benefit is restricted to delay treatment failure of glycemic control.

**8. In treatment-naïve, non-pregnant, asymptomatic adults recently diagnosed with T2DM in whom HbA**_**1c**_**is 7.5% to 9.0%, DUAL THERAPY is recommended to improve blood glucose control.** 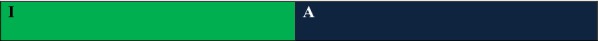


#### Summary of evidence

- The efficacy and safety of multiple dual therapies were compared with those of monotherapies in a meta-analysis of drug-naïve T2DM patients [11]. A total of 36 clinical trials in T2DM, longer than 12 weeks, in which initial therapy with two antidiabetic agents were compared to one agent were included. The primary endpoint was the change in HbA_1c_ from baseline. Compared with metformin monotherapy, an initial combination of DPP4i and metformin was associated with a significant decrease in HbA_1c_ by weighted mean difference (WMD − 0.44%, 95% CI − 0.57 to − 0.31, *p* < 0.001), without any increase in hypoglycemia nor in serious adverse effects, but with a small increase in body weight (WMD 0.38 kg, *p* < 0.001). Compared with metformin monotherapy, initial treatment combination of a sulfonylurea plus metformin resulted in significant decreases in HbA_1c_ (WMD − 0.68%, 95% CI − 0.86 to − 0.50, *p* < 0.001); however, it significantly increased the risk of hypoglycemia (RR 8.91, *p* = 0.02). Compared with metformin alone, initial combinations of a thiazolidinedione (TZD) plus metformin led to significant decreases in HbA_1c_ (WMD − 0.44%, 95% CI − 0.68 to − 0.19, *p* < 0.001) but also significantly increased the risk of hypoglycemia (RR 1.60, *p* = 0.03). Compared with metformin monotherapy, initial combinations of SGLT2i plus metformin led to significant decreases in HbA_1c_ (WMD − 0.47%, 95% CI − 0.58 to − 0.37, *p* < 0.001), but increased the risk of drug-related AEs (RR 1.45; *p* = 0.004). Compared with monotherapy, all initial combination therapies resulted in significantly reduced HbA_1c_ levels in treatment-naïve T2DM. Compared with metformin monotherapy, the initial combination of DPP-4i and metformin or SGLT2i plus metformin was associated with similar risks of hypoglycemia, but the initial combination therapies of sulfonylurea plus metformin and TZD plus metformin increased the risk of hypoglycemia.
- This panel recommends clinical judgment for choosing the appropriate drug, considering the level of HbA_1c_, the risk of hypoglycemia, tolerability and availability.

**9. In treatment-naïve, non-pregnant, asymptomatic adults recently diagnosed with T2DM without overt CVD or renal disease, DUAL THERAPY with metformin plus an AD1 is recommended for renal protection.** 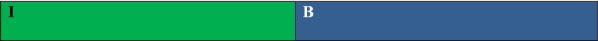


#### Summary of evidence

- Concerning renal protection with SGLT2i, the best evidence in T2DM with preserved renal function comes from the meta-analysis conducted by Neuen et al. [12]. This meta-analysis assessed the effects of SGLT2i on major kidney outcomes in patients with T2DM at different levels of GFR and determined the consistency of effect size across randomized clinical trials that reported effects on major kidney outcomes. The primary outcome was the composite of dialysis, transplantation, or death due to kidney disease. The authors used random-effects models to obtain summary relative risks (RRs) with 95% CIs and random-effects meta-regression to explore effect modification by subgroups of baseline GFR, albuminuria. Four studies met the inclusion criteria: EMPA-REG OUTCOME, CANVAS Program, CREDENCE and DECLARE–TIMI 58. From a total of 38,723 participants, 252 required dialysis or transplantation or died of kidney disease, 335 developed end-stage kidney disease, and 943 had acute kidney injury. SGLT2i reduced the risk of dialysis, transplantation, or death due to kidney disease (RR 0.67, 95% CI 0.52–0.86, *p* = 0.0019), an effect consistent across studies (I^2^ = 0%, p_heterogeneity_ = 0.53). In a subgroup analysis of patients with preserved eGFR (> 90 mL/min/1.73 m^2^), there were 12,167 patients with T2DM and 159 events occurred. The events were defined as: substantial loss of kidney function, end-stage kidney disease, or death due to kidney disease. The risk reduction in this subgroup was 0.37 (95% CI 0.21 to 0.63, *p* < 0.0001; I^2^ = 41.8%, p_heterogeneity_ = 0.18). The authors identified a proportional effect of SGLT2i despite attenuation of kidney function (p_trend_ = 0.073). These data provide indirect evidence supporting the use of SGLT2i in preventing major kidney outcomes in people with T2DM independently of baseline renal function, including in patients with preserved renal function.
- The SGLT2i trial with the greatest number of patients with preserved renal function was the DECLARE TIMI 58 trial [13]. This trial randomized 25,698 patients with T2DM at risk for atherosclerotic cardiovascular disease with GFR > 60 mL/min/1.73 m^2^ to receive either dapagliflozin or placebo for a median follow up of 4.2 years. The mean baseline eGFR was 85.4 mL/min/1.73 m^2^. A significant number of patients (47.6%) had eGFR > 90 mL/min/1.73 m^2^ and 45.1% had eGFR 60–90 mL/min/1.73 m^2^. Moreover 70.9% were normoalbuminuric (UACr < 30 mg/mmol). The secondary renal outcome in DECLARE was defined as a composite of 40% decrease in GFR to < 60 mL/min/1.73 m^2^, new end-stage renal disease, death from renal or cardiovascular causes, and/or death from any cause. This outcome was reduced by dapagliflozin (HR 0.76, 95% CI 0.67 to 0.87, *p* < 0.0001).
- The best evidence for a renal-protective effect of GLP-_1_RA in patients without CKD comes from REWIND RENAL [14]. This was a sub-analysis of renal outcomes in the REWIND study. REWIND RENAL compared dulaglutide and placebo for a median follow-up of 5.4 years in a multicenter, randomized, double-blind, placebo-controlled design. Originally, REWIND included patients with T2DM who had either a history of previous cardiovascular events or cardiovascular risk factors, with a large proportion of patients having normal renal function, and the mean GFR was 76.9 mL/min/1.73/m^2^. The mean urinary albumin-to-creatinine ratio (UACr) was 1.80 mg/mmol (95% CI 0.70 to 6.60). Of these patients, 65% had normoalbuminuria, 75% had eGFR > 60 mL/min/1.73 m^2^, and 47.5% had both eGFR > 60 mL/min/1.73 m^2^ and normoalbuminuria. The renal component of the microvascular outcome was defined as a composite of first occurrence of new albuminuria (UACR > 33.9 mg/mmol) and/or sustained decline in eGFR of 30% or more from baseline and/or onset of chronic renal replacement therapy. The renal outcome occurred in 17.1% of participants in the dulaglutide group versus 19.6% of participants in the placebo group (HR 0.85; 95% CI 0.77 to 0.93; *p* = 0.0004). These findings suggest that long-term use of dulaglutide was associated with reduced incidence of renal outcomes and better renal protection in people with T2DM without renal disease.
- Further evidence of the renal protective effect of GLP-_1_RA in T2DM without CKD comes from LEADER RENAL [15], a sub-analysis of secondary renal outcomes from the LEADER trial, an RCT comparing liraglutide against placebo, in which 9340 patients with T2DM were included and followed up for 3.8 years. Only 10% had microalbuminuria or proteinuria, 34.7% had normal eGFR (> 90 mL/min/1.73 m^2^), and 41.7% had only mild loss of renal function (GFR 60–89 mL/min/1.73 m^2^). The secondary renal outcome of LEADER—a composite of new-onset persistent macroalbuminuria, persistent doubling of serum creatinine, end-stage renal disease, or death due to renal disease—was observed in fewer participants receiving liraglutide versus placebo (HR 0.78; 95% CI 0.67 to 0.92; p = 0.003). This difference was attributable to a lower rate of new-onset persistent macroalbuminuria in the liraglutide group (HR 0.74; 95% CI 0.60 to 0.91; *p* = 0.004).
- This panel considered, however, that either an SGLT2i or a GLP_1_-RA should be used along with metformin in DUAL THERAPY. This is due to the finding that the majority of patients were on metformin in all trials: DECLARE (dapagliflozin), 81% [16]; EMPA-REG-OUTCOME (empagliflozin), 73.8% [17]; CANVAS (canagliflozin), 77% [18]; LEADER (liraglutide), 76% [14]; REWIND (dulaglutide), 81.3% [19]; and SUSTAIN-6 (semaglutide), 73% [20]. Thus, the panel considered that the effect of these agents in the aforementioned trials cannot be dissociated from the effects of metformin.

**10. In treatment-naïve, non-pregnant, asymptomatic adults recently diagnosed with T2DM without overt CV disease or renal disease, and in whom HbA**_**1c**_**is 7.5–9.0%, DUAL THERAPY with metformin plus an AD1 should be considered to reduce cardiovascular events.** 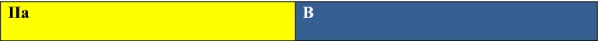


#### Summary of evidence

- In a meta-analysis [21] of seven randomized clinical trials comparing GLP-_1_RA vs. placebo, CV events (3P-MACE) and CV mortality were evaluated. A total of 27,977 patients on GLP_1_-RA and 28,027 in the placebo group were analyzed. For the 3P-MACE, the hazard ratio (HR) indicating benefit of GLP-_1_RA was 0.88 (95% CI 0.82 to 0.94, *p* < 0.0001), with a number needed to treat (NNT) of 75 in 3.2 years. For cardiovascular mortality, the benefit of GLP-_1_RA was also evident. The HR was 0.88 (95% CI 0.81 to 0.96, *p* < 0.003), and the NNT was 163 in 3.2 years.
- Post-hoc analysis of the LEADER trial [22] found that patients without CV events but with subclinical atherosclerosis (i.e., in primary prevention) benefit from liraglutide to the same extent as those who have had CV events. The panel considered that a large proportion of T2DM patients, even without previous CV events, may have significant subclinical atherosclerosis. Thus, it may be reasonable to use GLP-_1_RA or SGLT2i preferentially in high-risk patients.
- In the EXSCEL trial [23], the primary endpoint (3P-MACE) occurred in 839 patients receiving exenatide once weekly (11.4%; 3.7 events per 100 person-years) versus 905 patients in the placebo group (12.2%; 4.0 events per 100 person-years). Exenatide was non-inferior to placebo (HR 0.91; 95% CI 0.83 to 1.00; *p* < 0.001), but not superior (*p* = 0.06 for superiority).
- Regarding SGLT2i, a meta-analysis of randomized, placebo-controlled trials in patients with T2DM analyzed the effect of this class on CV outcomes. Three trials and 34,322 patients (60.2% with established ASCVD) were included. SGLT2i reduced MACE by 11% (HR 0.89; 95% CI 0.83 to 0.96; *p* = 0.0014). However, this benefit was only seen in patients with ASCVD (0.86; 95% CI 0.80 to 0.93), not in those without (1.00; 95% CI 0.87 to 1.16; *p* = 0.0501 for interaction) [24].

**11. Whenever an AD1 is not available, in treatment-naïve, non-pregnant, asymptomatic adults recently diagnosed with T2DM without known CV disease or renal disease, and in whom HbA**_**1c**_**is 7.5–9.0%, DUAL THERAPY including metformin plus any AD is recommended to improve blood glucose control.** 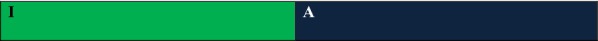


#### Summary of evidence

Adding DPP4i:

- Dual-therapy with DPP4i and metformin is efficacious and safe. A meta-analysis [25] assessing the long-term efficacy and safety of DPP4i combined with metformin compared to metformin alone in patients with T2DM included seven randomized clinical trials lasting at least 24 weeks. The decline in HbA_1c_ was greater with dual therapy. The difference was − 0.54% (95% CI − 0.63 to − 0.45), with no increase in hypoglycemia (HR 0.79; 95% CI 0.48 to 1.30).
- DPP4i have proven CV safety in the noninferiority CV outcome trials (CVOTs): TECOS (sitagliptin) [26], EXAMINE (alogliptin) [27], and CARMELINA (linagliptin) [28]. One exception is vildagliptin, which was not tested for safety in large CVOTs. Although the recent VERIFY [10] study indicated no signal of harm, it was not powered to detect CV safety. In SAVOR TIMI 53 (saxagliptin) [29], however, the frequency of HF hospitalization was higher in those receiving saxagliptin than in the placebo group.

Adding pioglitazone:

- Pioglitazone efficacy and safety was studied in patients with CV disease in the PROactive trial [30]. A prospective, randomized controlled trial including 5238 patients with T2DM who had evidence of macrovascular disease assigned to oral pioglitazone (15 to 45 mg) (*n* = 2605) or matching placebo (*n* = 2633), taken in addition to their glucose-lowering drugs. The primary endpoint was a composite of all-cause mortality, non-fatal myocardial infarction (including silent myocardial infarction), stroke, acute coronary syndrome, endovascular or surgical intervention in the coronary or leg arteries, and amputation above the ankle. The average time of observation was 34.5 months. The primary composite endpoint was not met (HR 0.90, 95% CI 0.80 to 1.02, *p* = 0.095); however, the main secondary endpoint (a composite of all-cause mortality, non-fatal myocardial infarction, and stroke, that is similar to MACE of more recent trials) was (HR 0.84, 95% CI 0.72 to 0.98, *p* = 0.027). Overall, safety and tolerability were good, with no change in the safety profile of pioglitazone identified (6% vs. 4% in the pioglitazone and placebo groups, respectively, were admitted to hospital due to heart failure; mortality rates from heart failure did not differ between groups).

Adding sulfonylureas:

- The safety of sulfonylureas in relation to CV outcomes was recently demonstrated in the CAROLINA [31] head-to-head randomized clinical trial (glimepiride versus linagliptin), in the TOSCA.IT [32] head-to-head trial (glimepiride versus pioglitazone), and in the ADVANCE [3] trial (gliclazide MR).
- In a meta-analysis of randomized clinical trials [33], CV safety was extended to glibenclamide as well. The panel considered that sulfonylureas are safe in relation to CV risk; however, they are associated with increased incidence of episodes of hypoglycemia. Prescription must thus be individualized for each patient. Among the sulfonylureas, gliclazide MR may be associated with a lower risk of hypoglycemia [34]. In the GUIDE randomized clinical trial, a large-scale (*n* = 845) head-to-head comparison of gliclazide MR and glimepiride, hypoglycemia occurred less frequently with gliclazide MR (3.7%) than with glimepiride (8.9%) (*p* = 0.003).

**12. In treatment-naïve, asymptomatic adults with T2DM and no overt CV or renal disease in whom HbA**_**1c**_**is > 9.0%, DUAL THERAPY including metformin and insulin-based therapy should be considered to improve blood glucose control.** 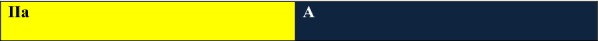


#### Summary of evidence

- A meta-analysis comparing CV and metabolic outcomes in insulin-based versus non-insulin-based glucose-lowering therapy included 19,300 adult patients across 18 RCTs. In 16 trials, insulin had superior efficacy in attaining blood glucose control (HR 0.20; 95% CI 0.28 to 0.11) and was associated with superior reductions in HbA_1c_. There was no significant between-group difference in risk of death from any cause or CV events. Baseline HbA_1c_ among all included studies ranged from 7.4 to 9.7%. The risk of hypoglycemia was higher among patients receiving insulin (RR 1.90; 95% CI 1.44 to 2.51). Non-insulin treatment was associated with a higher proportion of adverse drug reactions [35] (54.7% versus 45.3%, *p* = 0.044).
- Compared with oral ADs, early intensive insulin therapy in patients with newly diagnosed T2DM is associated with favorable impact on recovery and maintenance of beta-cell function, as well as protracted glycemic remission. A multicenter, randomized clinical trial compared the effects of transient intensive insulin therapy—continuous subcutaneous insulin infusion (CSII) or multiple daily injections (MDI)—versus oral antidiabetic agents on beta-cell function and diabetes remission. A total of 382 treatment-naïve patients with recently diagnosed T2DM were randomized to receive insulin or oral hypoglycemic agents for rapid initial correction of hyperglycemia. The mean HbA_1c_ at baseline was 9.5–9.8%. Treatment was stopped once normoglycemia had been achieved and remained stable for 2 weeks; patients were then followed on diet and exercise alone. Intravenous glucose tolerance tests were performed and glucose, insulin, and proinsulin levels were measured. The primary endpoint was duration of glycemic remission and remission rate at 1 year. Overall, more patients achieved target blood glucose control in the insulin groups than among those treated with oral ADs. The 1-year remission rate was significantly higher in the insulin groups (51.1% and 44.9% versus 26.7% with oral ADs; *p* = 0.0012). Beta-cell function, assessed by the HOMA-B and acute insulin response, also improved significantly after intensive therapy. The increase in acute insulin response was sustained in the insulin groups, but had declined significantly in the oral ADs group at 1 year in all patients who achieved remission [36].
- A meta-analysis of interventional studies to assess the effect of short-term intensive insulin therapy on the underlying pathophysiological defects of T2DM and to identify clinical predictors of remission (including HOMA-IR) analyzed 7 randomized and non-randomized trials with 839 participants. Pooled analysis showed an increase in HOMA-B from baseline after intensive insulin therapy (1.13, 95% CI 1.02 to 1.25), as well as a reduction in HOMA-IR (− 0.57, 95% CI − 0.84 to − 0.29). Four studies assessed glycemic remission (*n* = 559 participants). The proportion of patients with sustained remission was 66.2% at 3 months, 58.9% at 6 months, 46.3% at 12 months, and 42.1% after 24 months. The authors concluded that short-term intensive insulin therapy can improve underlying pathophysiology in early T2DM [37].

**13. In adult patients with T2DM who are symptomatic (polyuria, polydipsia, weight loss) and present with HbA**_**1c**_** > 9%, insulin-based therapy is recommended to improve blood glucose control.** 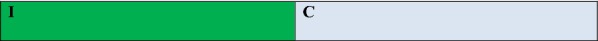


#### Summary of evidence

- The panel recommended the use of insulin-based therapy in T2DM patients with symptoms of hyperglycemia. There is general agreement that insulin-based therapy is needed when symptoms of insulin deficiency are present. This statement is based primarily on the pathophysiology of T2DM, plausibility, and clinical experience.

**14. In patients with T2DM without cardiovascular or renal complications, whose HbA**_**1c**_**remains above target despite dual therapy, TRIPLE THERAPY with metformin plus two AD1 is recommended to improve blood glucose control, renal protection and cardiovascular risk reduction.** 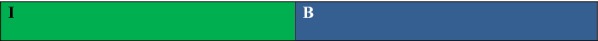


#### Summary of evidence

- The panel considered that, in general, triple therapy is effective and safe for improving blood glucose control. The majority of the cited studies indicate superior HbA_1c_-lowering efficacy with 3 than with 2 antidiabetic drugs.
- We found no trials directly comparing additive cardiovascular risk reduction or renal protection with a triple combination of SGLT2i and GLP-_1_RA plus metformin. However, SGLT2i have demonstrated reduction of renal outcomes in patients with preserved renal function [12], and GLP_-1_RA have demonstrated cardiovascular risk reduction in patients in primary prevention who had subclinical atherosclerosis [21]. Thus, the panel considered plausible that both effects can occur simultaneously with a combination of the two medications.
- Considering triple therapy with a combination of metformin/SGLT2i and GLP-_1_RA, the AWARD-10 [38] trial randomized 424 patients who were on SGLT2i and metformin to receive dulaglutide 1.5 mg (*n* = 142), dulaglutide 0.75 mg (*n* = 142), or placebo (*n* = 140). The primary objective was to test for superiority of dulaglutide versus placebo regarding change in HbA_1c_ from baseline at 24 weeks. HbA_1c_ was reduced further in patients receiving all three drugs (dulaglutide 1.5 mg: − 1.34%, SE 0.06; dulaglutide 0.75 mg: − 1.21%, SE 0.06) than in those receiving 2 drugs (placebo plus metformin/SGLT2i: − 0.54% (SE 0.06); *p* < 0.0001). Triple therapy improved blood glucose control significantly, with acceptable tolerability.
- The DURATION-8 study [39] was a 28-week, multicenter, double-blind, active-control trial of T2DM patients with HbA_1c_ 8–12% who were on metformin monotherapy. Patients (*n* = 695) were randomly assigned to receive exenatide plus dapagliflozin, exenatide plus placebo, or dapagliflozin plus placebo. The primary endpoint was change in HbA_1c_ from baseline to week 28. At 28 weeks, the change in HbA_1c_ was − 2.0% (95% CI − 2.2 to − 1.8) in the exenatide/dapagliflozin group, − 1.6% (− 1.8 to − 1.4) in the exenatide group, and − 1.4% (− 1.6 to − 1.2) in the dapagliflozin group. The combination of exenatide and dapagliflozin significantly reduced HbA_1c_ from baseline to week 28 compared with exenatide alone (− 0.4%; 95% CI − 0.6 to − 0.1; *p* = 0.003) or dapagliflozin alone (− 0.6%; 95% CI − 0.8 to − 0.3; *p* < 0.001), and was well tolerated.
- The combination of empagliflozin and linagliptin was examined as a second-line therapy in subjects with T2DM inadequately controlled on metformin in a double-blind randomized clinical trial [40]. Patients were randomized to empagliflozin plus linagliptin or each drug alone in different dosages as add-on to metformin for 52 weeks. The primary end-point was change in HbA_1c_ from baseline at week 24. At week 24, decreases in HbA_1c_ from a baseline of 7.90–8.02% were superior with empagliflozin/linagliptin than with empagliflozin 25 mg or linagliptin 5 mg alone as add-ons to metformin. Overall, 61.8% attained HbA_1c_ < 7% with the combination of empagliflozin 25 mg/linagliptin 5 mg, while only 32.6% did with empagliflozin 25 mg alone (OR 4.2, 95% CI 2.3 to 7.6, *p* < 0.001) and 36.1% with linagliptin 5 mg alone (OR 3.5, 95% CI 1.9 to 6.4, *p* < 0.001). Efficacy was maintained at week 52. The proportion of subjects with adverse events over 52 weeks was similar across treatment arms (68.6–73.0%), with no hypoglycemic AEs requiring assistance. The empagliflozin/linagliptin combination as second-line therapy for 52 weeks significantly reduced HbA_1c_ compared with the individual components, and was well tolerated.
- In an open-label clinical-trial [41], 106 patients recently diagnosed with T2DM were randomized to metformin/pioglitazone/exenatide (triple therapy) and 115 to metformin followed by sulfonylurea and insulin glargine (conventional therapy) with an HbA_1c_ target of < 6.5% for 2 years. Participants who received triple therapy had a greater reduction in HbA_1c_ level than those who received conventional therapy (5.95% versus 6.50%; *p* < 0.001). Despite lower HbA_1c_, participants on triple therapy experienced a 7.5-fold lower rate of hypoglycemia than patients on conventional therapy. Triple therapy was also associated with weight loss versus weight gain in those receiving conventional therapy (− 1.2 kg versus + 4.1 kg respectively; *p* < 0.01).
- A post hoc analysis of three randomized trials of sequential or concomitant add-on of dapagliflozin and saxagliptin to metformin [42] compared the safety of triple therapy (dapagliflozin + saxagliptin + metformin) versus dual therapy (dapagliflozin or saxagliptin + metformin). At 24 weeks, the incidence of any adverse events and serious adverse events was similar between the triple and dual therapy groups, as well as between the concomitant and sequential add-on groups. Urinary tract infections were more common in the sequential groups than in the concomitant groups; genital infections were reported only with sequential add-on of dapagliflozin to saxagliptin/metformin. Hypoglycemia occurred in < 2.0% of patients across all groups.
- A network meta-analysis [43] compared the efficacy of adding a third AD in patients with T2DM not well controlled (HbA_1c_ > 7%) by dual-therapy with metformin and a sulfonylurea. The meta-analysis included only randomized trials of at least 24 weeks’ duration. The primary outcomes were change in HbA_1c_, change in weight, and frequency of severe hypoglycemia. A total of 18 trials involving 4535 participants, with a mean duration of 31 weeks, were included. Compared with placebo, the drug classes did not differ regarding effect on HbA_1c_ level, with reductions ranging from − 0.70% (95% CI − 1.33% to − 0.08%) to − 1.08% (95% CI − 1.41% to − 0.77%). Weight gain was seen with insulin (2.84 kg; 95% CI 1.76 to 3.90 kg) and with thiazolidinediones (4.25 kg; 95% CI 2.76 to 5.66 kg), while weight loss was seen with GLP-_1_RA (− 1.63 kg; 95% CI − 2.71 to − 0.60 kg). Insulin caused twice as many severe hypoglycemic episodes than noninsulin ADs. No agent was superior to any other in terms of HbA_1c_ reduction. This meta-analysis did not test SGLT2i.

**15. In patients with T2DM without cardiovascular or renal complications, whose HbA**_**1c**_**remains above target despite triple therapy, QUADRUPLE THERAPY with metformin, two AD1 and one AD is recommended to improve blood glucose control.** 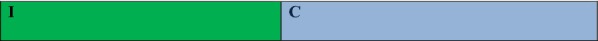


#### Summary of evidence

- Although this panel did not find evidence for using insulin exclusively as a fourth drug in quadruple therapy, there was consensus in the expert opinions for its use due to the efficacy and safety of insulin-based therapy.

**16. In patients with T2DM whose HbA**_**1c**_**remains above target despite triple therapy, QUADRUPLE THERAPY including combinations of metformin plus one AD1 and two AD or even metformin plus 3 AD or insulin based-therapy should be considered to improve blood glucose control.** 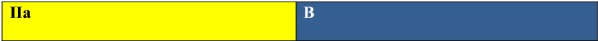


#### Summary of evidence

- Quadruple therapy was evaluated in an open-label observational trial [44] conducted in patients with T2DM not controlled (HbA_1c_ 7.5–12%) despite three different antidiabetic agents. The objective was to address the effectiveness and safety of adding empagliflozin or insulin glargine as a fourth agent in patients already on metformin, glimepiride and a DPP4i. A total of 268 patients were included: 142 on empagliflozin (25 mg/day) and 126 on insulin glargine. After 24 weeks, HbA_1c_ was significantly reduced from baseline by 1.5 ± 1.2% (*p* < 0.001) in the empagliflozin group and 1.1 ± 1.8% (*p* < 0.001) in the insulin group.
- Adverse effects occurred in 21.1% and 27.0% of subjects in the empagliflozin and insulin glargine groups, respectively. Adverse effects leading to treatment discontinuation were reported for 9 patients: 3 (2.1%) in the empagliflozin group and 6 (4.8%) in the insulin group. Hypoglycemic events were the most common adverse effects in both groups, and significantly higher (25.4% vs. 10.6%, *p* = 0.001) in the insulin versus empagliflozin groups, respectively. Therefore, quadruple therapy with metformin, a sulfonylurea, a DPP4i and SGLT2i may be considered effective and safe for treating T2DM.
- In a 26-week open-label trial [45], patients receiving GLP_1RA_ therapy (liraglutide once daily or exenatide twice daily) plus metformin alone or metformin plus pioglitazone and/or a sulfonylurea were randomly assigned to receive insulin degludec plus liraglutide once daily (*n* = 292) or to continue GLP_1RA_ therapy and oral ADs at the pre-trial dose (*n* = 146). At 26 weeks, superior HbA_1c_ reductions had been achieved with the insulin degludec/liraglutide combination (estimated treatment difference − 0.94%; *p* < 0.001).
- An open-label, prospective, 52-week study [46] was conducted in T2DM to compare the effectiveness and safety of adding empagliflozin 25 mg od or dapagliflozin 10 mg od as part of a quadruple therapy regimen for patients already on metformin, glimepiride and DPP4i and still inadequately controlled (HbA_1c_ 7.5–12.0%). The outcome measure was change in HbA_1c_. In total, 350 patients were enrolled with empagliflozin (*n* = 176) and dapagliflozin (*n* = 174), respectively. After 52 weeks, both groups showed significant reductions in HbA_1c_, but the reduction was greater in the empagliflozin group (*p* < 0.001). Safety profiles were similar in the two groups, demonstrating that quadruple therapy can be used effectively in patients with T2DM.
- In a 26-week open-label trial [45], patients receiving GLP-_1_RA therapy (liraglutide once daily or exenatide twice daily) plus metformin alone or metformin plus pioglitazone and/or a sulfonylurea were randomly assigned to receive insulin degludec plus liraglutide once daily (*n* = 292) or to continue GLP-_1_RA therapy and oral ADs at the pre-trial dose (*n* = 146). At 26 weeks, superior HbA_1c_ reductions had been achieved with the insulin degludec/liraglutide combination (estimated treatment difference − 0.94%; *p* < 0.001).

### Atherosclerotic cardiovascular disease (ASCVD) (Fig. 2)

**Treatment of choice:**

**Fig. 2:**
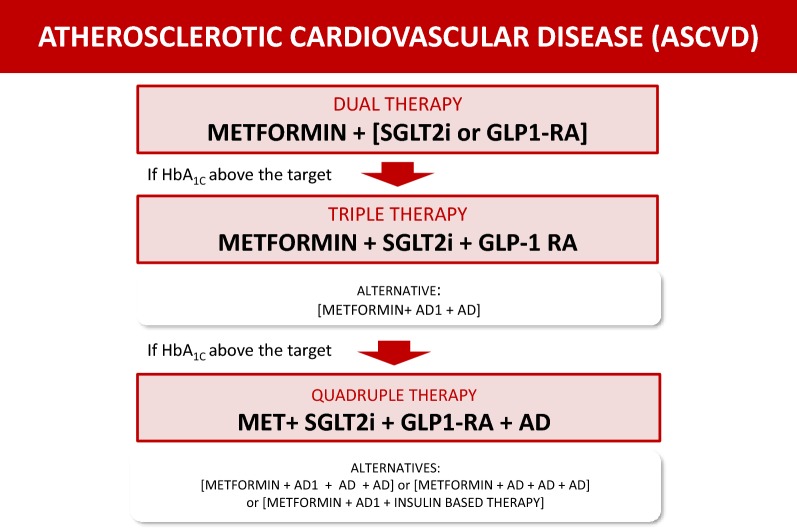
Decision support algorithm for treatment of hyperglycemia in patients with type 2 diabetes mellitus and atherosclerotic cardiovascular disease

**17. In patients with T2DM and clinical atherosclerosis (ASCVD), the combination of metformin with either an SGLT2 inhibitor or a GLP1-RA (AD1) is recommended to reduce cardiovascular events and to improve blood glucose control.** 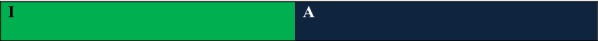


#### Summary of evidence

- In a meta-analysis [24] of three randomized, placebo-controlled CVOTs of SGLT2i in patients with T2DM, with or without ASCVD, the efficacy outcome was the classical 3P-MACE composite. The analysis included three trials and 34,322 patients (60.2% with established ASCVD). There were 3342 MACE and 2028 cardiovascular deaths. Overall, SGLT2i reduced 3P-MACE by 11% (HR 0.89; 95% CI 0.83 to 0.96; *p* = 0.0014). This benefit was driven by the EMPA-REG-OUTCOME (empagliflozin, HR 0.86, 95% CI 0.74 to 0.99) and CANVAS PROGRAM (canagliflozin, HR 0.82, 95% CI 0.72 to 0.95) trials. Benefit was only seen in the subgroup of patients with ASCVD. No heterogeneity in between-study variance was found across subgroups (Q = 0.94, *p* = 0.63, I^2^ = 0%). No effect was seen in patients without CV disease.
- In the EMPA-REG OUTCOME trial [17], T2DM patients with CV disease were assigned to receive either 10 mg or 25 mg of empagliflozin or placebo once daily. The primary outcome was 3P-MACE; CV mortality alone was assessed as a secondary outcome. A total of 7020 patients were treated for a median time of 3.1 years. 3P-MACE occurred in 10.5% in the pooled (10 + 25 mg) empagliflozin group and in 12.1% in the placebo group (HR 0.86; 95% CI 0.74 to 0.99; *p* < 0.001 for noninferiority, *p* = 0.04 for superiority). CV death rates were lower in the empagliflozin group (3.7% versus 5.9%; HR 0.62; 95% CI 0.49 to 0.77; *p* < 0.001), corresponding to a 38% relative risk reduction.
- In a meta-analysis [21] of seven randomized, placebo-controlled CVOTs of GLP-_1_RA enrolling a total of 56,004 participants, the overall hazard reduction for the primary outcome (3P-MACE) was 12% (HR 0.88; 95% CI 0.82 to 0.94; *p* < 0.0001). This benefit was driven by LEADER (liraglutide, HR 0.87, 95% CI 0.78 to 0.97, *p* = 0.015); SUSTAIN-6 (injectable semaglutide, HR 0.74, 95% CI 0.58 to 0.95, *p* = 0.016); HARMONY OUTCOMES (albiglutide, HR 0.78, 95% CI 0.68 to 0.90, *p* < 0.0001); and REWIND (dulaglutide, HR 0.88, 95% CI 0.79 to 0.99, *p* = 0.026). The overall risk of CV mortality was reduced to a similar extent (HR 0.88; 95% CI 0.81 to 0.96; *p* = 0.003), with this benefit driven by LEADER (liraglutide, HR 0.78, 95% CI 0.66 to 0.93) and PIONEER 6 (oral semaglutide, HR 0.49, 95% CI 0.27 to 0.92, *p* = 0.021).
- In the LEADER trial [47], 9340 T2DM patients were randomized to receive liraglutide or placebo. The median follow-up was 3.8 years. Overall, 82% of patients had established CV disease (31% with previous myocardial infarction, 15% with previous stroke, and 38% with a history of revascularization). Around 25% of patients had > 50% stenosis of coronary, carotid, or lower-limb arteries. Cardiovascular mortality occurred in fewer patients in the liraglutide group (4.7%) than in the placebo group (6.0%) (HR 0.78; 95% CI 0.66 to 0.93; *p* = 0.007). A post hoc analysis of the LEADER trial [22] assessed the CV outcomes in T2DM patients with or without history of myocardial infarction or stroke. Patients were stratified into three groups: (1) previous MI or stroke; (2) no CV events, but documented subclinical ASCVD; and (3) CV risk factors only. Liraglutide reduced the incidence of 3P-MACE compared to placebo in patients with previous CV events (15%; HR 0.85, 95% CI 0.73 to 0.99) and in those with subclinical ASCVD (14%; HR 0.76, 95% CI 0.62 to 0.94). Liraglutide did not reduce events in patients with CV risk factors alone.

**18. In T2DM patients with ASCVD and HbA**_**1c**_**above the target despite dual therapy with an AD1 and metformin, TRIPLE THERAPY with metformin and a combination of two AD1 (SGLT2i and GLP-**_**1**_**RA) is recommended to reduce cardiovascular events and improve glycemic control.** 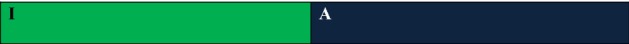


#### Summary of evidence

- Studies specifically designed to test whether triple therapy can reduce MACE in T2DM patients with ASCVD were not found in the literature. However, in the EMPA-REG OUTCOME trial [17], 49% of patients were on dual therapy before being randomized to empagliflozin. Thus, almost half of patients in whom cardiovascular events were significantly reduced received triple therapy. Considering the robust data in reducing 3P-MACE with both GLP-_1_RA and SGLT2i, as described above, this panel considered that the combination of both SGLT2i and GLP1-RA should be preferred among other antidiabetic agents, as they are also safe and effective for reducing blood glucose.

**19. In T2DM patients with ASCVD and HbA**_**1c**_**above target despite triple therapy, QUADRUPLE THERAPY in a combination of metformin, two AD1 and one AD is recommended to improve blood glucose control.** 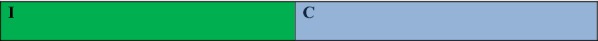


#### Summary of evidence

- Evidence from trials using exclusively quadruple therapy in T2DM patients with atherosclerotic cardiovascular disease is lacking. The best evidence available is described in statement 15 of this guideline, referring to quadruple therapy in the general patient with T2DM. This panel considered that the aforementioned evidence does overlap with patients with ASCVD, as high-risk patients were tested in individual trials for safety. This panel agrees that quadruple therapy is recommended whenever HbA_1c_ targets are not reached despite triple therapy, even in patients with ASCVD.

Alternative treatment in patients with ASCVD:

**20. Whenever AD1 is unavailable and HbA**_**1c**_**is 6.5–7.5%, metformin in MONOTHERAPY is recommended as the initial therapy to improve blood glucose control and reduce cardiovascular events in T2DM patients with clinical atherosclerosis (ASCVD).** 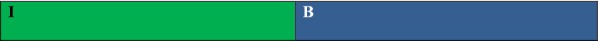


#### Summary of evidence

- A meta-analysis of clinical trials and observational studies [48] assessed the impact of metformin versus placebo and active comparators on mortality and cardiovascular events among T2DM patients, including sub-groups with coronary artery disease (CAD), to evaluate death from all causes, CV death, and incidence of CV events. The meta-analysis included 1,066,408 patients across 40 studies. Death from CV causes, death from any cause, and incidence of CV events were reduced among patients with CAD who received metformin, with HR 0.81 (95% CI 0.79 to 0.84, *p* < 0.00001); HR 0.67 (95% CI 0.60 to 0.75, *p* < 0.00001); and HR 0.83 (95% CI 0.78 to 0.89, *p* < 0.00001), respectively. A subgroup analysis showed that metformin reduced mortality from any cause in patients with a history of myocardial infarction (HR 0.79; 95% CI 0.68 to 0.92; *p* = 0.003) and in those with heart failure (HF) (HR 0.84; 95% CI 0.81 to 0.87). The incidence of CV events was also reduced among those with HF (HR 0.83; 95% CI 0.70 to 0.98).

Alternatives to AD1 in patients with ASCVD:

**21. Whenever an AD1 is unavailable and HbA**_**1c**_**is above 7.5%, despite metformin monotherapy, DUAL THERAPY with metformin and any AD is recommended to improve blood glucose control in patients with T2DM and clinical atherosclerosis (ASCVD).** 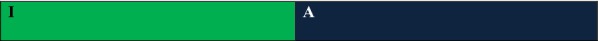


#### Summary of evidence

- The efficacy and safety of DPP-4i and pioglitazone in improving hyperglycemia in patients with ASCVD is well established in the TECOS (sitagliptin) [26], SAVOR-TIMI 53 (saxagliptin) [32], CARMELINA (linagliptin) [28], and PROactive (pioglitazone) [30] trials. The efficacy and safety of sulfonylureas in patients with ASCVD were confirmed in CAROLINA (glimepiride) [31], TOSCA.IT (glimepiride) [32], and ADVANCE (gliclazide MR) [3], as well as in a meta-analysis of clinical trials [35].

**22. Alternatively, if only one AD1 is available and HbA**_**1c**_**is above 7.5% despite dual therapy in T2DM patients with ASCVD, TRIPLE THERAPY with metformin plus an AD1 and any other AD is recommended to improve blood glucose control.** 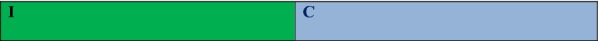


#### Summary of evidence

- A network meta-analysis of 176,310 participants across 236 trials [49] found that SGLT2i and GLP-_1_RA were associated with significantly lower rates of death from any cause as compared to control. SGLT2i (absolute risk reduction − 0.9%; HR 0.78; 95% CI 0.68 to 0.90) and GLP-_1_ agonists (absolute risk reduction, − 0.5%; HR 0.86; 95% CI 0.77 to 0.96) were associated with lower mortality, while DPP4i were not associated with significant reductions in death from any cause (absolute risk reduction, 0.1%; HR, 1.02; 95% CI 0.94 to 1.11). Mortality was lower in patients receiving SGLT2i or GLP-_1_RA than in those receiving DPP4i, placebo, or no treatment.

### Heart failure (Fig. 3)

**Treatment of choice:**

**Fig. 3:**
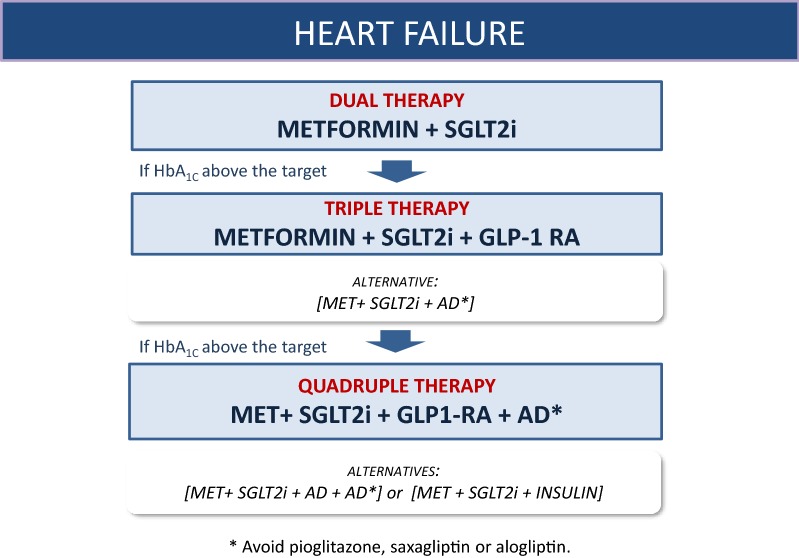
Decision support algorithm for treatment of hyperglycemia in patients with type 2 diabetes mellitus and heart failure

**23. In patients with T2DM and heart failure (HF) with reduced ejection fraction (< 40%), combined therapy including metformin and an SGLT-2i is recommended to reduce cardiovascular mortality, HF hospitalizations, and to improve blood glucose control.** 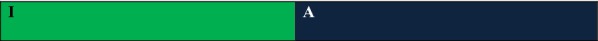


#### Summary of evidence

- In a systematic review and meta-analysis [24] of three randomized, placebo-controlled CVOTs of SGLT2i in 34,322 patients with T2DM, SGLT2i reduced the risk of CV death or HF hospitalization by 23% (HR 0.77; 95% CI 0.71 to 0.84, *p* < 0.0001), with a similar benefit in patients with and without a history of HF. The magnitude of benefit depended on with baseline renal function; greater reductions in HF hospitalization (*p* = 0.0073 for interaction) and lesser reductions in progression of renal disease (*p* = 0.0258 for interaction) were observed in patients whose kidney disease was more severe at baseline. SGLT2i reliably reduce the rate of hospital admission for HF regardless of existing ASCVD or history of HF.
- The DAPA-HF clinical study [50] randomized 4744 patients with symptomatic HF with reduced ejection fraction (HfrEF) to receive dapagliflozin 10 mg once daily or placebo. The primary endpoint was CV death or HF events (hospitalization or urgent visit for HF). Secondary endpoints were hierarchically tested in sequence: composite of CV death or hospitalization for HF; composite of recurrent hospitalizations for HF or CV death; change from baseline in total Kansas City Cardiomyopathy Questionnaire (KCCQ) symptom score at 8 months; composite of ≥ 50% sustained decline in GFR, end-stage renal disease, or renal death; and death from any another cause. Dapagliflozin reduced the primary endpoint of CV death or HF events by 26% (11.6% vs. 15.6%; HR 0.74, 95% CI 0.65 to 0.85, *p* < 0.0001) after a mean follow-up of 18 months. All components of the first composite endpoint contributed to the treatment effect, and the effects were generally consistent across subgroups, including patients with T2DM and those without diabetes (*p* = 0.7965 for interaction).
- A prospective observational study [48] was conducted to assess the effect of starting metformin on the prognosis of patients with newly diagnosed HF and new-onset T2DM treated with a “contemporary medical regimen” for 9 years. A total of 1519 patients were enrolled; mean age was 71 years, 53.8% were women, and 51.3% had preserved systolic function. Over a median follow-up of 57 months, 1045 patients (68.8%) died and 1344 (88.5%) were hospitalized for decompensation of HF. There were no cases of lactic acidosis attributable to metformin use. Metformin was associated with decreased mortality (HR 0.85, 95% CI 0.82 to 0.88), largely driven by a lower CV mortality (HR 0.78, 95% CI 0.74 to 0.82), as well as a lower hospitalization rate (HR 0.81, 95% CI 0.79 to 0.84). Nevertheless, metformin was not associated with an improved prognosis of HF in patients with a mean HbA_1c_ ≤ 7.0%.
- In all three CVOTs of SGLT2i (EMPA-REG OUTCOME, CANVAS, and DECLARE-TIMI 58), metformin was the cornerstone of treatment, in 74%, 77%, and 82% of patients, respectively. Considering that SGLT2i was used as an add-on to metformin, this panel considered that the effects of SGLT2i cannot be separated from that of metformin. Therefore, dual therapy is recommended in treatment-naïve T2DM patients with HF [16–18].

**24. In patients with T2DM and HF with reduced ejection fraction (< 40%) whose HbA**_**1c**_**is above target despite dual therapy with metformin and an SGLT2i, TRIPLE THERAPY by adding a GLP-**_**1**_**RA should be considered to reduce the risk of HF-related hospitalization.** 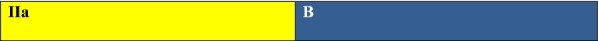


#### Summary of evidence

- A meta-analysis of seven randomized placebo-controlled trials [21], including 56,004 high-risk patients with T2DM, reported the effects of GLP-_1_RA on hospital admission for HF as a secondary outcome. GLP-_1_RA treatment reduced HF admissions by 9% (HR 0.91, 95% CI 0.83 to 0.99; *p* = 0.028). The reduction was not robust; the number needed to treat (NNT) was 312 (95% CI 165 to 2810) over 3.3 years. Although an additive effect of SGLT2i and GLP-_1_-RA in reducing cardiovascular outcomes has not yet been proved, this panel considered that, if further improvement of blood glucose control is needed, adding a GLP-_1_RA may be plausible and interesting in T2DM patients with HF.

**25. In T2DM patients with low ejection-fraction HF and HbA**_**1c**_**above target despite triple therapy, QUADRUPLE therapy including metformin, an SGLT2 inhibitor, a GLP-1 RA and a fourth antidiabetic agent (AD) or insulin-based therapy is recommended to improve blood glucose control.** 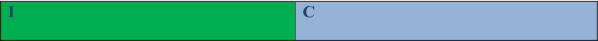


#### Summary of evidence

- Evidence referring exclusively to use of quadruple therapy in patients with T2DM and HF was not found in the literature. The best evidence available is described in statement 15 of this guideline, referring to quadruple therapy for the general patient with T2DM. This panel agrees that quadruple therapy is recommended whenever HbA_1c_ targets are not reached despite triple therapy, even in patients with ASCVD. However, based on expert opinion, a combination of agents with proven CV safety (i.e., which do not increase risk of HF) is reasonable.

**26. Saxagliptin, alogliptin, and pioglitazone are not recommended as AD in patients with HEART FAILURE with reduced ejection fraction due to the risk of worsening HF.** 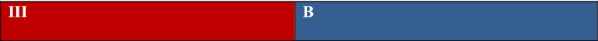


#### Summary of evidence

- In the SAVOR-TIMI 53 noninferiority trial [29], T2DM patients at risk of CV events were randomly assigned to receive saxagliptin or placebo, and followed for a median of 2.1 years. The primary efficacy and safety endpoint was the classic 3P-MACE. Of 16,492 patients randomized, more were hospitalized for HF in the saxagliptin group than in the placebo group (3.5% versus 2.8%; HR 1.27; 95% CI 1.07 to 1.51; *p* = 0.007). The number needed to harm (NNH) was 143, with HF occurring early in the first year of treatment. Patients with high NT-proBNP levels, CKD, or previous HF were at increased risk.
- In the EXAMINE [51] noninferiority trial, patients with T2DM having experienced acute coronary syndrome in the previous 15 to 90 days were randomly assigned to receive alogliptin or placebo plus standard care for T2DM and CV disease prevention. The prespecified exploratory endpoint was an extension of MACE: all-cause mortality, non-fatal myocardial infarction, nonfatal stroke, urgent revascularization due to unstable angina, and hospital admission for HF. Overall, 5380 patients were assigned to alogliptin (*n* = 2701) or placebo (*n* = 2679), and followed for a median of 533 days. The endpoint occurred in 16.0% of patients in the alogliptin group versus 16.5% in the placebo group (HR 0.98, 95% CI 0.86 to 1.12). HF-related hospitalization was the first event in 3.1% of patients taking alogliptin versus 2.9% in the placebo group (HR 1.07, 95% CI 0.79 to 1.46). This similar event rate notwithstanding, the panel recommends that alogliptin be avoided in patients with established HF.
- A systematic review and meta-analysis [52] of seven double-blind RCTs compared the risk of development of HF in patients given thiazolidinediones (either rosiglitazone or pioglitazone) versus controls. The main outcome was development of congestive HF and risk of CV death. Of 20,191 included patients with either prediabetes or T2DM, 360 developed congestive HF events (214 in thiazolidinediones and 146 on comparators), which suggested a class effect of thiazolidinediones. Compared with controls, patients given these agents had increased risk of developing HF across a wide background of cardiac risk (relative risk 1.72, 95% CI 1.21 to 2.42, *p* = 0.002). Conversely, the risk of CV death was not increased with thiazolidinediones (RR 0.93, 95% CI 0.67 to 1.29, *p* = 0.68).
- The PROactive Study [30] was a prospective randomized clinical trial of 5238 patients with T2DM and macrovascular disease. Patients were randomized to receive pioglitazone or placebo. The primary endpoint was an expanded MACE composite including death from any cause, nonfatal myocardial infarction, stroke, acute coronary syndrome, endovascular or surgical intervention in the coronary circulation or lower-limb arteries, and above-ankle amputation. The mean observation time was 34.5 months. The primary endpoint occurred similarly in patients in the pioglitazone group and patients in the placebo group (HR 0.90, 95% CI 0.80 to 1.02, *p* = 0.095). Patients receiving pioglitazone experienced more HF episodes than on placebo (11% versus 8%, *p* < 0.0001). There was also a higher number of HF episodes not needing hospital admission (5% vs. 3%; *p* = 0.003) and HF episodes requiring hospital admission (*p* = 0.007) in pioglitazone-treated patients versus placebo. However, there was no difference in the rate of fatal HF.

Alternative treatment:

**27. In T2DM patients with low ejection-fraction HF and HbA**_**1c**_**above target despite dual therapy with METFORMIN and an SGLT2 inhibitor, the institution of TRIPLE THERAPY by adding (alternatively to GLP**_**1**_**-RA) an AD or insulin-based therapy is recommended to improve blood glucose control.** 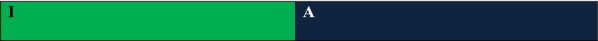


#### Summary of evidence

Adding a sulfonylurea:

- In the UKPDS trial [53], HF rates were not increased among patients who received sulfonylureas as compared with the conventional treatment group (3.0% vs. 3.3%, HR 0.91, 95% CI 0.54 to 1.52).
- An observational study [54] investigated all-cause mortality associated to sulfonylureas (SU) in patients with HF. Patients who were hospitalized for the first time due to HF in 1997–2006, alive 30 days after discharge, and on monotherapy with a specific type of SU were followed for a mean of 744 days. There were 1097 patients on glimepiride; 1031 on glibenclamide; 557 on glipizide; 251 on gliclazide; and 541 on tolbutamide. During the observation period, 2242 patients (64%) died. Compared to gliclazide, which was considered the reference, the risk of death was similar among all types of SU: glimepiride (HR 1.10, 95% CI 0.92 to 1.33); glibenclamide (HR 1.12, 95% CI 0.93 to 1.34), glipizide (HR 1.14, 95% CI 0.93 to 1.38) and tolbutamide (HR 1.04 (0.85–1.26). Significant differences in mortality risk among SU in patients with HF were deemed unlikely.

Adding insulin:

- Insulin has a dose-dependent anti-natriuretic effect, and causes weight gain and mild edema at physiologic concentrations. We found no controlled trials addressing safety of insulin in patients with clinically established HF or at high risk of HF. In UKPDS 33 [2], there was no difference in HF rates between patients receiving insulin and those on sulfonylureas.
- Insulin glargine, a long-acting insulin analogue, was studied in the ORIGIN trial [55]. A sub-analysis showed that insulin glargine has a neutral effect on both initial and recurrent hospitalizations for HF. The trial randomized 12,537 patients with prediabetes or diabetes to either insulin glargine or placebo. All were at high cardiovascular risk. However, people with more severe HF—New York Heart Association (NYHA) class 3 or 4—were excluded. There were no differences between groups in hospitalization for HF (HR 0.90, 95% CI 0.77 to 1.05) over the 2.5 years of follow-up [56]. The position of this panel is that insulin can be used as a safe option to control blood glucose in patients with HF. However, close monitoring is advisable in patients with unstable HF.

Adding a DPP4i:

- The TECOS [26] noninferiority trial was designed to assess the efficacy and safety of sitagliptin in 14,671 subjects with CV disease. The study found that sitagliptin did not increase hospitalization for HF as compared to placebo (HR 1.00, 95% CI 0.83 to 1.20) during a 3-year follow-up period. CV mortality was similar between sitagliptin and placebo (22.4% versus 23.1%), as well as all-cause mortality, after hospitalization (29.8% versus 28.8%).
- CARMELINA [28] was a randomized, placebo-controlled, multicenter noninferiority trial conducted among adults with type 2 diabetes to test linagliptin (*n* = 3494) against placebo (*n* = 3485) as add-on over usual care. Hospitalization for HF (an exploratory cardiovascular outcome) occurred in 209 of 3494 patients in the linagliptin group (6.0%) and in 226 of 3485 patients in the placebo group (6.5%). The absolute difference in incidence rate was − 0.27 (95% CI − 0.82 to 0.28), which was nonsignificant (HR 0.90; 95% CI 0.74 to 1.08; *p* = 0.26).
- We did not find CVOTs conducted to assess the CV safety of vildagliptin. However, a retrospective meta-analysis [57] did not find any significant increase in risk of HF in vildagliptin-treated patients.

### Chronic kidney disease (CKD) (Fig. 4)

**Mild to moderate CKD** (eGFR 30–60 mL/min/1.73 m^2^ or eGFR 30–90 mL/min/1.73 m^2^ with albuminuria)

**Fig. 4:**
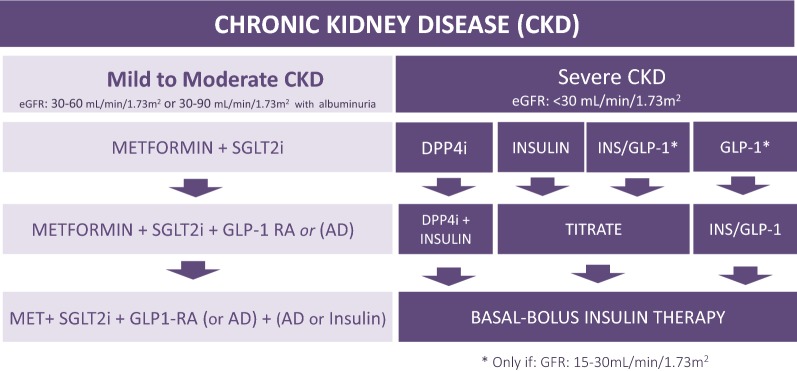
Decision support algorithm for treatment of hyperglycemia in patients with type 2 diabetes mellitus and chronic kidney disease

**28. In T2DM patients with mild to moderate CKD, DUAL THERAPY with metformin and an SGLT2i is recommended to attenuate loss of renal function, prevent end-stage renal disease, reduce mortality due to renal causes, and to improve blood glucose control.** 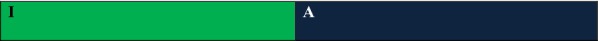


#### Summary of evidence

- The CREDENCE study [58] randomly assigned patients with T2DM with HbA_1c_ 6.5–12% and CKD (calculated eGFR 30–90 mL/min/1.73 m^2^ and albuminuria > 300–5000 mg/g) to receive either canagliflozin (100 mg/day) or placebo. Metformin was used by 57.8% of patients. A total of 4401 patients underwent randomization (mean age 63 years, 33.9% female). The mean HbA_1c_ was 8.3% and the mean eGFR was 56.2 mL/min/1.73 m^2^. CV disease was present in 50% of patients. The median urinary albumin was 927 mg/g. The primary outcome was a composite of end-stage kidney disease (dialysis for at least 30 days, kidney transplantation, or eGFR < 15 mL/min/1.73 m^2^ for at least 30 days, doubling of serum creatinine from baseline or death from renal of cardiovascular disease). Over a median follow-up of 2.62 years, the primary outcome occurred in significantly fewer patients in the canagliflozin group than in the placebo group (43.2 versus 61.2/1000 patients-year, respectively; HR 0.70, 95% CI 0.59 to 0.82, *p* = 0.00001).
- In a sub-analysis of EMPA-REG OUTCOME [17], 4124 T2DM patients with GFR > 30 mL/min/1.73 m^2^ were assigned to either empagliflozin or placebo once daily. The secondary renal outcomes (all prespecified) included incident or worsening nephropathy (progression to macroalbuminuria, doubling of serum creatinine, initiation of renal replacement therapy, or renal death) and incident albuminuria. Incident or worsening nephropathy occurred in 12.7% of patients in the empagliflozin group versus 18.8% in the placebo group (HR 0.61; 95% CI 0.53 to 0.70; *p* < 0.001). Doubling of serum creatinine occurred in 1.5% of patients receiving empagliflozin and in 2.6% of those given placebo (a significant relative risk reduction of 44%). Renal replacement therapy was initiated in 0.3% in the empagliflozin group and in 0.6% in the placebo group (a 55% lower relative risk). The rate of incident albuminuria was similar in the two groups.
- Dapagliflozin can reduce progression of kidney disease compared with placebo in T2DM. In the DECLARE–TIMI 58 trial [16], T2DM patients with ASCVD, multiple risk factors, and creatinine clearance of at least 60 mL/min/1.73 m^2^ were randomized to receive dapagliflozin or placebo. The prespecified secondary cardiorenal composite outcome was “a sustained decline of at least 40% in GFR rate, end-stage renal disease (dialysis, kidney transplantation, or confirmed sustained GFR < 15 mL/min/1.73 m^2^), or death from renal or CV causes”. The median follow-up was 4.2 years. At baseline, of 17,160 patients, 47.6% had eGFR > 90 mL/min/1.73 m^2^, 45.1% had GFR 60–90 mL/min/1.73 m^2^, and 7.4% had GFR < 60 mL/min/1.73 m^2^. The secondary outcome was significantly reduced by dapagliflozin versus placebo (HR 0.76, 95% CI 0.67 to 0.87, *p* < 0.0001). After excluding CV mortality, the renal-specific outcome had a HR of 0.53 (95% CI 0.43 to 0.66, *p* < 0.0001). The decline in eGFR rate was attenuated in 46% of patients. The risk of ESRD or renal death was lower in the dapagliflozin group than in the placebo group (0.1% versus 0.3%; HR 0.41, 95% CI 0.20 to 0.82; *p* = 0.012). Both the cardiorenal and renal-specific composite outcomes were improved by dapagliflozin versus placebo in several prespecified subgroups, including those defined by baseline eGFR and presence or absence of established ASCVD. The mean decrease in eGFR was greater in the dapagliflozin group than in the placebo group 6 months after randomization; however, this decline had equalized by 2 years, and at 3 and 4 years the mean decrease in eGFR was less in the dapagliflozin group than in the placebo group. Metformin is recommended in T2DM and CKD, as it was used by 74% of patients in EMPA-REG OUTCOME trial [17], 58% of patients in the CREDENCE study [58], 76% of participants in CANVAS [18], and 81% of participants in DECLARE-TIMI 58 [16]. The panel considered that the effects of SGLT2i in these studies cannot be dissociated from those of metformin.
- The evidence suggests that metformin can be used safely in patients with serum creatinine < 1.5 mg/dL. Since serum creatinine may overestimate renal function, calculation of the estimated GFR is preferred. Metformin should not be initiated when eGFR is < 45 mL/min/1.73 m^2^. When eGFR is 45–30 mL/min/1.73 m^2^, the dosage should be reduced. Metformin should be stopped when eGFR falls below 30 mL/min/1.73 m^2^ [59].
- A meta-analysis [12] of RCTs included studies which reported the effects of SGLT2i on “major kidney outcomes” (a composite of chronic dialysis, renal transplantation, or renal death) in people with T2DM. Four studies met the inclusion criteria, assessing three SGLT2i: empagliflozin (EMPA-REG OUTCOME), canagliflozin (CANVAS Program and CREDENCE), and dapagliflozin (DECLARE–TIMI 58). SGLT2i substantially reduced the risk of dialysis, transplantation, or renal death (HR 0.67, 95% CI 0.52 to 0.86, *p* = 0.0019). The effect was consistent across studies (I^2^ = 0%, *p* = 0.53).

**29. In patients with T2DM and mild to moderate CKD whose HbA**_**1c**_**remains above the target despite dual therapy, TRIPLE THERAPY with metformin, SGLT2i and a GLP-**_**1**_**RA is recommended to reduce renal outcomes and improve glycemic control.** 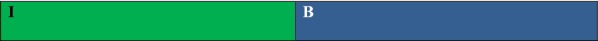


#### Summary of evidence

Adding a GLP-_1_RA:

- The LEADER RENAL [15] sub-study was a prespecified sub-analysis of secondary renal outcomes of the original LEADER trial, in which patients were randomized to receive liraglutide or placebo. In this sub-analysis, the secondary outcome was a composite of new-onset persistent macroalbuminuria, persistent doubling of serum creatinine, ESRD, or renal death. A total of 4668 patients were randomized to liraglutide and 4672 to placebo. Most patients were male (64.7%); the mean age was 64.4 years, and 82% had CV disease. The mean GFR was 80 mL/min/1.73 m^2^, but 20.7% had GFR 30–59 mL/min/1.73 m^2^ and 2.4% had a GFR < 30 mL/min/1.73 m^2^. Micro- and macroalbuminuria were present in 26.3% and 10.5% of patients, respectively. In 76% of patients, liraglutide was used as an add-on of metformin. Fewer patients in the liraglutide group experienced a renal outcome than in the placebo group (HR 0.78; 95% CI 0.67 to 0.92; *p* = 0.003).
- A sub-analysis of the REWIND study [14], comparing dulaglutide against placebo in T2DM, was conducted to examine secondary renal outcomes. The composite renal outcome was defined as “development of macroalbuminuria (development of UACR > 33.9 mg/mmol in people with a lower baseline concentration), a sustained 30% or greater decline in eGFR, or new chronic renal replacement therapy (comprising dialysis or renal transplantation)”. A total of 9901 participants were randomized (1:1) to receive dulaglutide or placebo. The median duration of follow-up was 5.4 years. The mean HbA_1c_ was 7.3%, and the eGFR, 76.9 mL/min/1.73 m^2^. Around 35% of participants had albuminuria and 22.2% had a eGFR < 60 mL/min/1.73 m^2^. The renal outcome occurred in 17.1% of patients in the dulaglutide group versus 19.6% of those in the placebo group (HR 0.85; 95% CI 0.77 to 0.93; *p* = 0.0004). The largest effect was seen for new-onset macroalbuminuria (HR 0.77, 95% CI 0.68 to 0.87, *p* < 0.0001). Numeric reductions were also seen in sustained decline of 30% or more in eGFR (HR 0.89, 95% CI 0.78 to 1.01, *p* = 0.066) and new chronic renal replacement therapy (HR 0.75, 95% CI 0.39 to 1.44, *p* = 0.39), despite no statistical significance.
- The efficacy of triple therapy in glycemic control of T2DM patients with moderate-to-severe CKD was evaluated in the AWARD-7 study [60], a multicenter, randomized, open-label, non-inferiority trial designed to compare dulaglutide versus insulin glargine in patients with T2DM who were already on insulin plus an oral AD. Approximately 90% of patients had eGFR between 30 and 60 mL/min/1.73 m^2^; 32–39% had microalbuminuria and 44% had macroalbuminuria. Patients who were on insulin or insulin plus an oral AD were randomized to receive dulaglutide (1.5 mg or 0.75 mg) or insulin glargine. Insulin lispro was also added and titrated. The trial lasted 52 weeks. The primary outcome was HbA_1c_ at 26 weeks. Secondary outcomes included eGFR and UACr. A total of 577 patients were randomized to dulaglutide and insulin glargine. Dulaglutide produced blood glucose control similar to that achieved with insulin glargine and slowed the decline in GFR. This study demonstrates that dulaglutide is safe and effective in patients with moderate-to-severe CKD.

Alternative treatment:

**30. In T2DM patients with mild to moderate CKD and HbA**_**1c**_**above target despite dual therapy, TRIPLE THERAPY with metformin, SGLT2 and an alternative AD (replacing GLP1-RA) is recommended to improve blood glucose control.** 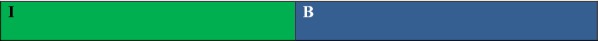


#### Summary of evidence

- The efficacy and safety of triple therapy with an AD in T2DM with chronic kidney disease was addressed in studies using DPP-4, pioglitazone and sulfonylureas.

Adding DPP4i:

- The CARMELINA trial evaluated linagliptin in [28] a placebo-controlled, multicenter, non-inferiority randomized clinical trial that included 6979 T2DM patients with high CV and renal risk. Patients had either a GFR between 45 and 75 mL/min/1.73 m^2^ along with UACr > 200 mg/g or a GFR between 15 and 45 mL/min/1.73 m^2^ regardless of UACr. Around 40% of patients were on dual therapy at baseline and received triple therapy. The median duration of follow-up was 2.2 years. The mean age was 65.9 years, mean eGFR was 54.6 mL/min/1.73 m^2^, and most of patients had eGFR between 30 and 60 mL/min/1.73 m^2^. Regarding albuminuria, 41.9% had UACr 30–300 mg/g and 38% had UACR > 300 mg/g. This study evaluated the impact of linagliptin versus standard care on incidence of the primary outcome (3P-MACE). The primary outcome (CV death, non-fatal myocardial infarction, or nonfatal stroke) was similar in both groups group (HR 1.02, 95% CI 0.89 to 1.17); however, the outcome was significant for non-inferiority, indicating safety (*p* < 0.001). Considering the renal outcomes (end-stage renal disease, death due to renal failure, or a sustained decrease from baseline of at least 40% in eGFR), there were also no differences (HR 1.04; 95% CI 0.89 to 1.22; *p* = 0.62). The rates of adverse events, serious adverse events, and adverse events leading to discontinuation were not different between linagliptin and placebo. Linagliptin is considered safe in renal failure.
- The safety of sitagliptin in patients with type 2 diabetes and moderate eGFR ≥ 30 to < 50 mL/min or severe renal insufficiency eGFR < 30 mL/min/1.73 m^2^ including patients with end-stage renal disease (ESRD) on dialysis was assessed in a 54-week, randomized, double-blind, parallel-group study, patients with baseline HbA1 between 6.5 and 10%. Sitagliptin group included 65 patients and placebo 26 patients. At 54 weeks, patients continuously treated with sitagliptin had a mean change (95% CI) from baseline in HbA(1c) of − 0.7% (− 0.9, − 0.4) [61].
- In the COMPOSIT-R clinical trial [62], patients were randomized to receive either sitagliptin or dapagliflozin. The trial included 614 T2DM patients with HbA_1c_ 7.0–9.5% and chronic kidney disease (eGFR 60–90 mL/min/1.73 m^2^), who were on metformin alone or metformin plus a sulfonylurea. The mean eGFR at baseline was 79.4 ± 11.3 mL/min/1.73 m^2^. Around 30% of patients were on dual therapy (metformin plus a sulfonylurea). After 24 weeks, the change in HbA_1c_ from baseline was greater with sitagliptin (− 0.51%, 95% CI − 0.60 to − 0.43) than dapagliflozin (− 0.36%, 95% CI − 0.45 to − 0.27). The difference was − 0.15% (95% CI − 0.26 to − 0.04) in favor of sitagliptin (*p* = 0.006). Overall, adverse effects occurred in 48.9% in the sitagliptin group, a rate similar to that of the dapagliflozin group (51.9%). The incidence of hypoglycemia was 15–16% among patients who were on triple therapy including metformin plus sulfonylurea plus sitagliptin. No serious adverse event or deaths were reported with triple therapy.

Adding pioglitazone:

- A meta-analysis [63] evaluated the efficacy and safety of thiazolidinediones, including pioglitazone and rosiglitazone, in the treatment of T2DM patients with renal impairment. Nineteen RCTs were included, covering 1818 participants, with a mean age ranging from 43.4 to 71.1 years, mean baseline HbA_1c_ of 6.9 to 9.2%, and mean follow-up of 24 weeks. Of the 19 RCTs, one (5.3%) enrolled patients who had undergone renal transplantation, five (26.3%) enrolled dialysis patients, and 13 (68.4%) included patients with mild to moderate renal impairment. Fourteen trials (73.7%) used pioglitazone as the intervention, four (21.1%) used rosiglitazone, and one (5.3%) used both. Thiazolidinediones were not associated with increased risk of all-cause mortality (RR 0.40, 95% CI 0.08 to 2.01) and did not increase the risk of HF (RR 0.64, 95% CI 0.15 to 2.66, I^2^ = 0%); however, they did increase the risk of edema significantly as compared to control (RR 2.96, 95% CI 1.22 to 7.20).
- A small efficacy and tolerability trial [64] randomized 93 patients with T2DM and CKD (defined as eGFR < 60 mL/min/1.73 m^2^ or albuminuria), of whom 30% were stage II, 32% were stage III, and 27% were stage IV, to pioglitazone 15 mg (standard-dose) or 7.5 mg (low-dose) for 24 weeks. Efficacy and tolerability were assessed. The mean change in HbA_1c_ did not differ between the standard-dose and low-dose groups (1.1 ± 1.6 and − 1.4 ± 1.5, *p* = 0.543, respectively). Standard-dose pioglitazone was associated with greater increases in body weight, fat mass, total body mass, water, and extracellular water compared to the low-dose regimen. Compared to patients in the 7.5-mg group, those receiving 15-mg pioglitazone experienced significant, though modest, weight gain (3.5 ± 3.2 versus 0.2 ± 4.4 kg; mean difference between groups, 3.3 kg, 95% CI 1.3 to 5.2). No major adverse effects (including hypoglycemia, congestive HF, and abnormal liver function) were identified. This study indicated that low-dose pioglitazone has similar efficacy while promoting less weight gain than standard-dose pioglitazone in patients with CKD.

Adding sulfonylureas:

- The safety of sulfonylureas was evaluated in the CAROLINA trial [31], a head-to-head, active-controlled, randomized trial that assessed the impact of linagliptin versus glimepiride on CV outcomes in high-risk patients (many with chronic kidney disease). The eGFR (mL/min/1.73 m^2^) was 60–89 in 58%, 30–59 in 19%, and 15–29 in 0.4% of participants. The primary outcome was time to first occurrence of a 3P-MACE event (CV death, nonfatal MI, or nonfatal stroke), with the aim of establishing the noninferiority of linagliptin versus glimepiride. A primary outcome event occurred in 356 of 3023 patients (11.8%) in the linagliptin group and 362 of 3010 (12.0%) in the glimepiride group (HR 0.98, 95.47%CI 0.84–1.14; *p* < 0.001 for non-inferiority). Thus, linagliptin met the noninferiority criterion but not the superiority criterion (*p* = 0.76). The incidence of adverse events was similar in the linagliptin and in glimepiride groups. Hypoglycemia, as expected, was increased in the glimepiride group: 10.6% in the linagliptin group and in 37.7% in the glimepiride group (HR, 0.23 [95% CI 0.21–0.26]).

**31. In T2DM patients with mild to moderate CKD and HbA**_**1c**_**above target despite triple therapy, QUADRUPLE THERAPY including metformin, SGLT2i, GLP**_**1**_**-RA and a fourth antidiabetic agent (AD) or insulin-based therapy is recommended to improve blood glucose control.** 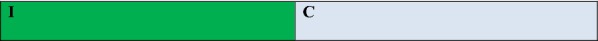


#### Summary of evidence

- Although we did not find significant efficacy evidence for QUADRUPLE therapy in T2DM patients with mild to moderate renal failure, the panel considered that this strategy is necessary to lower blood glucose in some patients and is reasonably safe when eGFR is between 30 and 90 mL/min/1.73 m^2^, a stage of CKD in which most agents can be used, provided that their dosages are adjusted when appropriate. Special attention is warranted with metformin, which should be replaced when the eGFR falls below 30 mL/min/1.73 m^2^. Sulfonylureas also demand caution due to an increased risk of hypoglycemia in this population.

Adding a DPP4i:

- The CARMELINA trial [28] evaluated exploratory outcomes including the progression of albuminuria in patients on linagliptin and placebo. Among 6979 participants, the mean eGFR was 54.6 mL/min/1.73 m^2^, and 80.1% had a UACR > 30 mg/g. Progression of albuminuria (from normo- to micro- or macroalbuminuria, or from micro- to macroalbuminuria) was less frequent in the linagliptin group (35.3%; 21.4 per 100 person-years) than in the placebo group (38.5%; 24.5 per 100 person-years). The absolute difference in incidence was − 3.18 (95% CI − 5.44 to − 0.92; HR 0.86, 95% CI 0.78 to 0.95, *p* = 0.003).
- Saxagliptin decreased albuminuria in T2DM patients with normoalbuminuria, microalbuminuria, and macroalbuminuria, regardless of baseline eGFR. The SAVOR RENAL analysis [65] studied renal outcomes in 16,492 patients with T2DM who had been randomly assigned to receive saxagliptin or placebo. The median duration of follow-up was 2.1 years. At baseline, 9696 subjects (58.8%) were normoalbuminuric, 4426 (26.8%) were microalbuminuric (ACR 30–300 mg/g), and 1638 (9.9%) were macroalbuminuric (ACR > 300 mg/g). Saxagliptin therapy was associated with less deterioration in ACR from baseline at the end of the study (*p* = 0.021, *p* < 0.001). At 2 years, the difference in mean ACR change between saxagliptin and placebo was 219.3 mg/g (*p* = 0.033) for an estimated eGFR > 50 mL/min/body surface area (BSA), 2105 mg/g (*p* = 0.011) for eGFR 30–50 mL/min/BSA, and 2245.2 mg/g (*p* = 0.086) for eGFR < 30 mL/min/BSA. Changes in ACR did not correlate with changes in HbA_1c_. The change in eGFR was similar between the saxagliptin and placebo arms.

Adding a sulfonylurea:

- The ADVANCE [3] trial randomly assigned 11,140 patients with T2DM to either intensive or standard glucose control, defined as the use of gliclazide MR plus other drugs as needed to achieve an HbA_1c_ target < 6.5%. Overall, 1434 of 5571 patients in the intervention group (27%) and 1423 of 5569 in the standard-of-care group (26.7%) had microalbuminuria at baseline. After a median 5 years of follow-up, new-onset microalbuminuria had occurred in 1318 patients (23.7%) in the intensive group versus 1434 (25.7%) in the standard-of-care group (HR, 0.91; 95% CI 0.85 to 0.98; *p* = 0.02).

Adding pioglitazone:

- The effect of thiazolidinediones on albuminuria in T2DM was evaluated in a meta-analysis [66] of 15 randomized controlled trials. A total of 2860 T2DM patients with baseline normo- or microalbuminuria, using both rosiglitazone and pioglitazone compared with placebo or other ADs, were evaluated. Overall, in participants with normo- and microalbuminuria, thiazolidinedione therapy was associated with significant reductions in urinary albumin excretion. In studies of pioglitazone, the weighted mean difference of proportional change between the pioglitazone and control groups was 16.2% (95% CI 20.8 to 11.6). The overall mean difference of the change in urine UACR between the thiazolidinedione and control groups was 24.8% (95% CI 39.6 to 10.0]. Thiazolidinediones, especially pioglitazone, reduce urinary albumin and protein excretion significantly in patients with T2DM.

**Severe CKD** (eGFR < 30 mL/min/1.73 m^2^ and hemodialysis)

**32. In T2DM patients with severe renal failure and HbA**_**1c**_**above target, insulin-based therapy is the recommended choice to improve blood glucose control.** 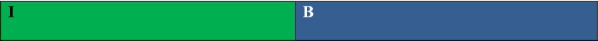


#### Summary of evidence

- Insulin glargine is safe and effective in T2DM patients with severe renal failure [67], yielding rapid HbA_1c_ reductions with a stable half-life and longer duration of action. In a small non-randomized study, 89 patients with T2DM and CKD (mean eGFR 34.1 ± 11.5 mL/min/1.73 m^2^), who were poorly controlled or experienced frequent hypoglycemia on oral ADs or NPH insulin, were prescribed insulin glargine at bedtime. The dose was started at 0.1 unit/kg and titrated to the desired target. At 4 months of follow-up, HbA_1c_ had declined from 8.4% ± 1.6 to 7.7% ± 1.2 (*p* < 0.001). Body mass index was unaffected (*p* = 0.96). Mild symptomatic hypoglycemia was experienced by 12.5% of patients. No other adverse events were reported.

**33. In T2DM patients with severe renal failure and HbA**_**1c**_**above target, either a DPP4 inhibitor or a GLP-**_**1**_**RA (if eGFR 15–30** **mL/min/1.73** **m**^**2**^**) may be considered to improve blood glucose control.**
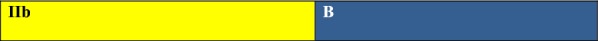


#### Summary of evidence

- The DPP4i class (sitagliptin, vildagliptin, alogliptin, saxagliptin and linagliptin) was also tested in small studies in T2DM patients undergoing hemodialysis, and safety should be confirmed in larger studies.
- In a small trial [68], 64 patients with T2DM were randomized to sitagliptin (in the reduced dosage of 25 mg/daily) and 65 to glipizide 2.5 mg/daily. There were 28 patients (43%) with eGFR < 30 mL/min/1.73 m^2^. After 54 weeks, the mean reduction in HbA_1c_ level from baseline was 0.72% (95% CI 0.95% to 0.48%) in the sitagliptin group and 0.87% (95% CI 1.11% to 0.63%) in the glipizide group. The incidence of symptomatic hypoglycemia was 6.3% in the sitagliptin group vs. 10.8% in the glipizide group (difference, 4.8%; 95% CI 15.7% to 5.6%). Severe hypoglycemia did not occur in the sitagliptin group vs. 7.7% in glipizide group (difference, 7.8%; 95% CI 17.1% to 1.9%). Sitagliptin monotherapy was effective and well tolerated in patients undergoing hemodialysis.
- Vildagliptin 50 mg once daily was evaluated in a 2-year open-label trial [69] including 32 patients with T2DM on hemodialysis. Changes in glycated albumin (GA) and dry weight were evaluated. GA was significantly reduced by 2.6 ± 0.6%, from 22.4 ± 0.6% at baseline to 19.8 ± 0.4% at 2 years. After 2 years of vildagliptin therapy, 15 (46.9%) of 32 patients achieved a GA level of < 20%. Dry weight changed slightly, with an increase of 1.3 ± 0.8 kg at 2 years. No adverse drug reactions related to treatment with vildagliptin were seen.
- In a small non-randomized safety trial [70], 16 patients with T2DM undergoing hemodialysis received alogliptin 6.25 mg for 2 years. Baseline serum creatinine was 10.6 ± 1.0 mg/dL. Mean HbA_1c_ dropped from 7.1 to 5.8% after treatment. None of the patients exhibited significant adverse effects, such as hypoglycemia. One patient experienced a drug-related rash. Four patients withdrew from this study during the treatment period.
- The effects of monotherapy with linagliptin 5 mg in 21 patients with T2DM undergoing hemodialysis was examined in a 6-month non-randomized trial [71]. Linagliptin was administered daily. GA dropped from 21.3% ± 0.6% to 18.0% ± 0.6% over the 6-month treatment period, and body weight did not change. None of the patients experienced hypoglycemia.
- Saxagliptin was studied in a sub-analysis of the SAVOR-TIMI 53 trial [72] according to baseline renal function. Patients with T2DM at risk of cardiovascular events were stratified by renal function. There were 336 patients with severe renal impairment (eGFR < 30 mL/min/1.73 m^2^) who were randomized to receive either saxagliptin or placebo. The primary endpoint was the time to first event of a composite of CV death, myocardial infarction (MI), or ischemic stroke. The major secondary endpoint included the primary composite plus hospitalization for heart failure, coronary revascularization, or unstable angina. After a median duration of 2 years, saxagliptin did not change the risk of the primary and secondary composite endpoints compared with placebo, irrespective of renal function (*p* = 0.19 for interactions). The relative risk of hospitalization for heart failure with saxagliptin was similar (*p* = 0.43 for interaction) in patients with GFR > 50 mL/min/1.73 m^2^ (HR 1.23, 95% CI 0.99 to 1.55), GFR 30–50 mL/min/1.73 m^2^ (HR 1.46, 95% CI 1.07 to 2.00), and in patients with GFR < 30 (HR 0.94, 95% CI 0.52 to 1.71). The median HbA_1c_ at 1 year was lower compared to placebo in saxagliptin-treated patients with severe renal impairment (7.1% vs. 7.7%, *p* = 0.002). At least one adverse event occurred in 152 (88%) saxagliptin-treated patients with severe renal impairment compared with 126 (77%) patients treated with placebo (*p* = 0.006), with no significant difference in severe adverse events.
- Data for the use of GLP-_1_RAs in T2DM with severe renal failure (< 30 mL/min/1.73 m^2^) are derived from subsets of larger trials that included a very small number of patients, such as 2.5% in LEADER RENAL (liraglutide) [15], 2.5% in SUSTAIN-6 (injectable semaglutide) [20], and 1% in REWIND RENAL (dulaglutide) [14]. Thus, data on the safety of GLP-_1_RA in this population is limited.

## Conclusions

In non-pregnant adults with type 2 diabetes, the recommended HbA_1c_ target is below 7%. Higher levels are recommended in frail older adults and patients at higher risk of hypoglycemia. Lifestyle modification is recommended at all phases of treatment. In recent diagnosed patients without cardiovascular or renal complications, metformin in monotherapy is the first choice of treatment when HbA_1c_ is 6.5–7.5%. Optionally, metformin along with a DPP4 inhibitor may be considered to reduce failure in controlling blood glucose. When HbA1c is 7.5–9.0%, dual therapy, including metformin a first line antidiabetic drug AD1 (SGLT2i or GLP-1RA) is recommended, due to their cardiovascular and renal benefits. If an AD1 is unaffordable, other antidiabetic drugs (AD) may be used. Triple or quadruple therapy should be considered when HbA1c remains above target despite dual therapy. In patients with clinical atherosclerosis, the combination of metformin plus one AD1 independently of HbA1c level is also recommended to reduce cardiovascular events. In the stable patient with low ejection fraction heart failure (< 40%) and glomerular filtration rate (eGFR) > 30 mL/min/1.73 m^2^, metformin plus an SGLT2i is recommended to reduce cardiovascular mortality, heart failure hospitalizations and to improve blood glucose control. In patients with mild to moderate diabetes-associated chronic kidney disease (CKD) (eGFR 30–60 mL/min/1.73 m^2^ or eGFR 30–90 mL/min/1.73 m^2^ with albuminuria > 30 mg/g), the combination of metformin and a SGLT2i is recommended to attenuate loss of renal function, reduce albuminuria and improve blood glucose control. In patients with severe renal failure (eGFR < 30 mL/min/1.73 m^2^), insulin-based therapy is recommended to improve blood glucose control. Alternatively, GLP-1RA, DPP4i, gliclazide MR and pioglitazone may also be considered to reduce albuminuria. In conclusion, the current evidences support individualizing anti-hyperglycemic treatment for T2DM according to their cardiovascular and renal status.

## Data Availability

Data sharing is not applicable to this article as no datasets were generated or analyzed during the current study.

## Abbreviations

AD1: Antidiabetic agents with proven cardiovascular or renal benefit
AD: Antidiabetic agents
ASCVD: Atherosclerotic cardiovascular disease
CKD: Chronic kidney disease
CVD: Cardiovascular disease
DM: Diabetes mellitus
eGFR: Estimated glomerular filtration rate
HF: Heart failure
T2DM: Type 2 diabetes mellitus
SGLT2I: Sodium–glucose transporter 2 inhibitor
GLP-_1_RA: Glucagon-like peptide-1 receptor agonist
